# DXA‐Measured Total and Regional Fat‐to‐Lean Mass Ratio and Mortality Risk in Chinese Older Adults: A 20‐Year Prospective Study

**DOI:** 10.1002/jcsm.70351

**Published:** 2026-07-13

**Authors:** Yafei Wu, Ting Zhang, Shuyi Li, Jason Leung, Timothy Kwok

**Affiliations:** ^1^ Department of Medicine and Therapeutics The Chinese University of Hong Kong Hong Kong SAR China; ^2^ Department of Geriatrics Ren Ji Hospital, Shanghai Jiao Tong University School of Medicine Shanghai China; ^3^ Jockey Club Centre for Osteoporosis Care and Control The Chinese University of Hong Kong Hong Kong SAR China

**Keywords:** dual‐energy x‐ray absorptiometry, fat‐to‐lean mass ratio, mortality, older adults

## Abstract

**Background:**

The association of fat‐to‐lean mass ratio (FLR) with mortality remains unclear in older adults. This study aimed to investigate the sex‐specific associations of total and regional FLR with mortality in older Chinese adults.

**Methods:**

This analysis included 2000 men (mean age: 72.4 ± 5.0 years) and 2000 women (mean age: 72.6 ± 5.4 years) aged ≥ 65 years from the Hong Kong OS cohort. Total and regional (trunk, abdominal, arms, legs) FLRs were measured using DXA at baseline. Mortality was ascertained via Hong Kong death registry. Multivariable Cox and Fine–Gray competing risk models were used to assess the associations of FLR with all‐cause, cardiovascular (CVD) and cancer mortality, with results presented as hazard ratios (HRs) and 95% confidence intervals (CIs). C‐index was used to quantify the discriminative ability of FLR measures and BMI in estimating mortality risk.

**Results:**

Over a median follow‐up of 18.2 years (IQR: 11.6–20.7), 2446 deaths (CVD: 511, cancer: 644) occurred. The 1‐, 10‐ and 20‐year survival rates were 0.990, 0.753 and 0.354 for men, and 0997, 0.858 and 0.500 for women. In men, FLR was inversely associated with all‐cause mortality when analysed continuously for whole‐body (HR per SD increase: 0.94, 95% CI: 0.89–1.00), trunk (HR: 0.92, 0.87–0.97), abdominal (HR: 0.92, 0.86–0.97) and arm FLR (HR: 0.94, 0.89–1.00), with all *p* < 0.05. In categorical analyses, men in the lowest tertile (T1) of whole‐body, trunk, abdominal and arm FLR had significantly higher mortality risk compared to those in the middle tertile (HRs > 1, all *p* < 0.05). In women, trunk (HR per SD increase: 0.94, 0.88–1.00, *p* = 0.041) and abdominal FLRs (HR: 0.91, 0.85–0.97) were inversely associated with all‐cause mortality, with T1 of abdominal FLR showing increased risk (HR: 1.18, 1.01–1.37) compared to T2. Restricted cubic splines revealed L‐shaped associations for most FLR measures (*p*‐nonlinearity < 0.05), except leg FLR in women (*P*‐nonlinearity = 0.060). For cause‐specific mortality, no significant associations were observed for CVD mortality in either sex, only leg FLR was inversely associated with cancer mortality in men (HR per SD increase: 0.89, 0.80–0.99; HR for T1 vs. T2: 1.29, 1.00–1.66, both *p* < 0.05), but not in women. All FLR measures showed higher discriminative ability than BMI for mortality prediction (all *p* < 0.01).

**Conclusions:**

In Chinese older adults, lower FLR was associated with higher all‐cause mortality in an L‐shaped relationship for both sexes, but showed no significant association with CVD mortality. Lower leg FLR was associated with increased cancer mortality only in men. FLR measures were more predictive than BMI for mortality risk stratification.

## 1 Introduction

Global demographic shifts, marked by population ageing and a concurrent increase in obesity, are intensifying the related disease burden [1]. In epidemiological studies, body mass index (BMI) has been the standard tool for classifying obesity and estimating mortality risk [2]. However, a key drawback of BMI is its inability to differentiate between fat mass and muscle mass, two physiologically distinct compartments that vary widely among individuals even at identical BMI values [3]. Body composition offers a more meaningful reflection of metabolic health than BMI, as it distinguishes between fat and muscle. Accumulating evidence suggests that the balance between fat and muscle may be critical in evaluating cardiometabolic risk [4]. Thus, the fat‐to‐muscle mass ratio (FMR) has gained attention as a composite indicator integrating these two counteracting components and has been shown to be associated with various health outcomes [5, 6, 7, 8].

To date, only a limited number of prospective studies have investigated the relationship between FMR and mortality and the findings remain inconsistent. For instance, Yu et al. identified J‐shaped relationships between whole‐body FMR and all‐cause mortality. They found both extremely low and high FMR were related to elevated mortality among younger men (< 50 years) and older women (≥ 60 years) [9]. Expanding on this, Xu et al. examined the association of both total and regional FMRs with all‐cause mortality and revealed distinct patterns: U‐shaped for whole‐body and trunk FMR, L‐shaped for leg FMR and J‐shaped for arm FMR [10]. Another investigation assessed the relationship between FMR and CVD mortality in individuals free of CVD at baseline and the results revealed that significant positive associations were predominantly observed for trunk and arm FMRs in male participants [8]. Despite these critical findings, important knowledge gaps persist. First, these associations were largely drawn from western populations (e.g., the UK Biobank), and studies specifically in older adults are limited. However, the ageing process involves a significant redistribution of fat and muscle, marked by the accumulation of visceral fat and a concurrent reduction in muscle mass [11]. Therefore, the generalizability of the findings to older people, particularly Chinese populations, warrants further investigation. Second, the predominant use of bioelectrical impedance analysis (BIA) for muscle mass estimation in prior studies may introduce bias. Although practical for large‐scale settings, BIA is generally considered less accurate than the dual‐energy X‐ray absorptiometry (DXA), as its measurements are more susceptible to variations in hydration status and rely on population‐specific predictive equations [12]. In contrast, DXA provides a more precise and direct assessment of body composition, including fat mass and lean mass. Although DXA‐derived lean mass includes non‐muscle components such as water and connective tissue, it is highly correlated with muscle mass, particularly appendicular lean mass [13] and is widely used in epidemiological studies.

To address the above limitations, we employed DXA‐derived fat‐to‐lean mass ratio (FLR) to investigate its association with long‐term mortality in Chinese older adults, utilizing data from the Mr. OS and Ms. OS (Hong Kong) cohort, a community‐based prospective study followed for up to 20 years. We evaluated sex‐specific associations of total and regional (trunk, abdominal, arms, legs) FLR with all‐cause, cardiovascular (CVD) and cancer mortality. Our findings are expected to inform targeted strategies in geriatric populations.

## 2 Methods

### 2.1 Study Design and Participants

**FIGURE 1:**
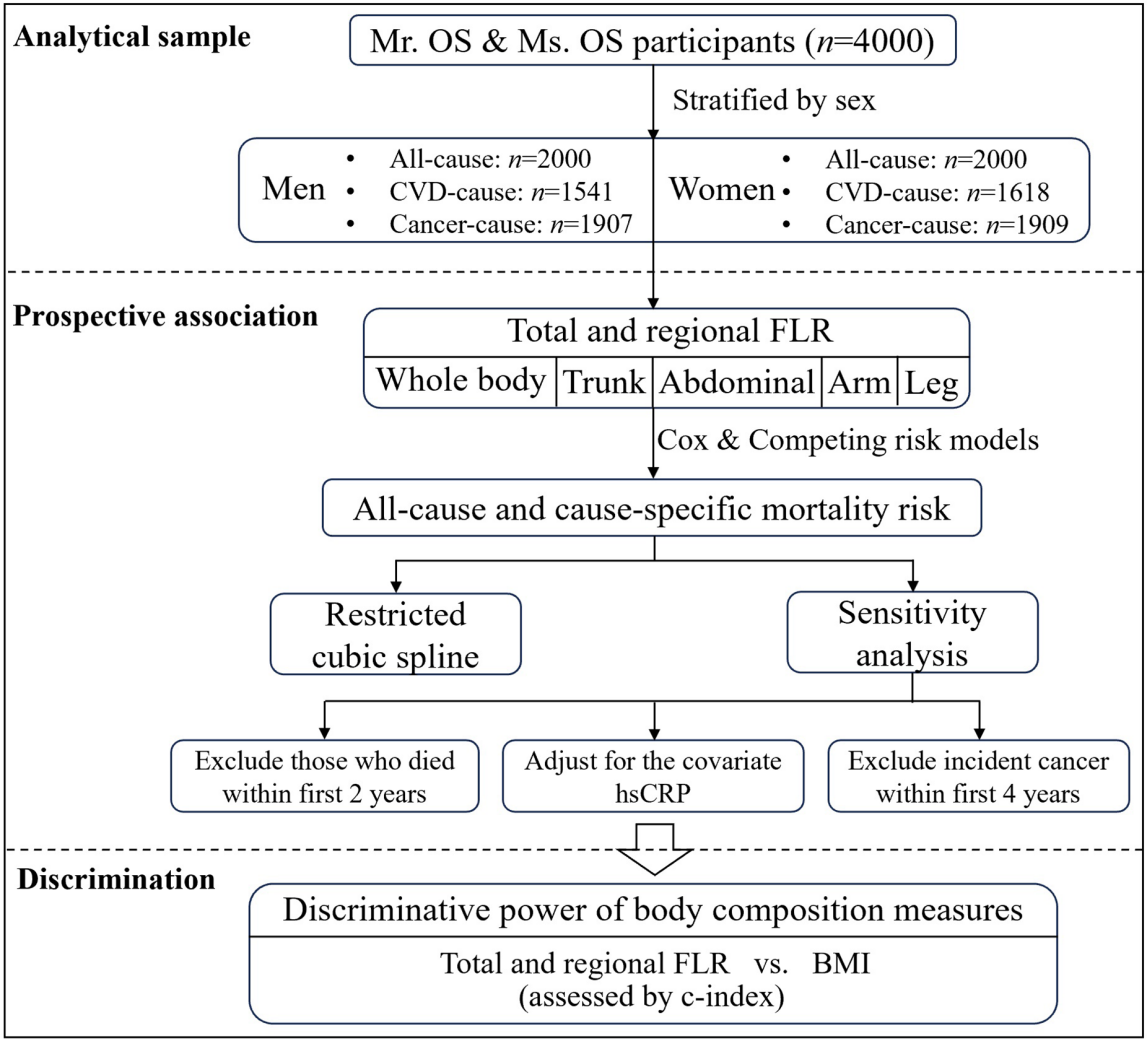
Flowchart for selection of the study sample from the Mr. OS and Ms. OS study. Abbreviation: FLR, fat‐to‐lean mass ratio.

This prospective study utilized data from the Mr. OS and Ms. OS (Hong Kong) cohort, an ongoing community‐based investigation of ageing in Chinese adults. The baseline survey was conducted between August 2001 and December 2003, recruiting a total of 2000 men and 2000 women aged 65 years or older, with follow‐up continuing to the present. The study was carried out in accordance with the Declaration of Helsinki and was approved by the Clinical Research Ethics Committee of The Chinese University of Hong Kong. All participants provided informed consent. More details about this cohort can be found in previous publications [14]. In the current study, participants who had complete DXA measurements (2000 men and 2000 women) were included for all‐cause mortality analysis. For cause‐specific mortality analysis, we excluded participants with missing cause of death and those who had CVD or cancer at baseline. This resulted in final analytical samples of 1541 men (excluding 7 without death causes and 452 with baseline CVD) and 1618 women (excluding 2 without death causes and 380 with baseline CVD) for CVD mortality and 1907 men (excluding 7 without death causes and 86 with baseline cancer) and 1909 women (excluding 2 without death causes and 89 with baseline cancer) for cancer mortality (Figure 1).

### 2.2 Body Composition Measures

At baseline survey, data on body composition including fat mass (FM) and lean mass (LM) were collected by trained healthcare technicians using DXA by a Hologic QDR 4500 W device (Waltham, Massachusetts, USA). Daily quality assurance and calibration were performed using a Hologic body composition step phantom at least three times per week and the maximum coefficient of variation for fat mass and lean mass is 1.47% and 0.84%, respectively. In this analysis, the fat‐to‐lean mass ratio (FLR) was calculated as fat mass divided by lean mass. We evaluated both total (whole‐body) and regional (trunk, abdominal, arms and legs) FLR. The regional FLR for each segment was derived by dividing the FM of that region by the LM of the same region. To ensure sufficient events in each group and to explore potential nonlinear relationships, all continuous FLR measures were also categorized into tertiles (T1‐T3). The middle tertile (T2) was used as the reference, as participants in this group were significantly younger and the restricted cubic spline analysis suggested the lowest mortality risk around the T2 range.

### 2.3 All‐Cause and Cause‐Specific Mortality

Mortality status and the underlying cause of death were ascertained through linkage with the Hong Kong Death Registry up to October 2023 (median follow‐up: 18.2 years, IQR: 11.6–20.7 years). Causes of death were classified according to the International Classification of Diseases, Tenth Revision (ICD‐10). For this analysis, deaths from CVD (ICD‐10 codes: I00‐I99) and cancer (ICD‐10 codes: C00‐C97) were the cause‐specific outcomes of interest. Person‐years were calculated from the baseline date to either the date of death or the date of October 2023, whichever came first.

### 2.4 Covariates

Potential confounders were selected based on established findings [8, 9, 10]. At baseline, trained interviewers administered standardized questionnaires to collect comprehensive information on covariates. These included demographic and lifestyle factors such as age (years), educational level (illiterate, primary or below, secondary or above), smoking status (non‐smoker, ex‐smoker, current smoker), alcohol consumption (yes/no) and physical activity level assessed using the Physical Activity Scale for the Elderly (PASE, with higher scores indicating greater physical activity) [15]. We also considered self‐reported physician‐diagnosed chronic conditions, including diabetes, hypertension, cardiovascular diseases (stroke, myocardial infarction, angina, congestive heart failure) and cancer, as well as medications use (antihypertensive and cholesterol‐lowering medications, both yes/no) to account for overall health status. Total or regional absolute lean mass was also adjusted. Only one value for alcohol consumption was missing and was imputed using the mode.

### 2.5 Statistical Analyses

Given the well‐established sexual dimorphism in body composition, all analyses were stratified by sex in current analyses [16]. Baseline characteristics of the participants were summarized by tertiles of whole‐body FLR. Continuous variables were presented as mean and standard deviation (SD) and categorical variables as number and percentages (%). Differences across tertiles were tested using one‐way analysis of variance (ANOVA) or the chi‐square test, as appropriate.

The associations between FLR (both as a continuous variable per SD increase and as tertiles) and all‐cause mortality were evaluated using multivariable Cox proportional hazards regression models. The proportional hazards assumption was tested using Schoenfeld residuals and no violation was observed. For cause‐specific mortality (CVD and cancer), we employed Fine–Gray subdistribution hazard models to account for competing risks. All models were adjusted for the predefined covariates. Further, the association between continuous FLR and all‐cause mortality was visualized using restricted cubic splines (with knots at the 10th, 50th and 90th percentiles) [17]. The potential non‐linearity was tested using likelihood ratio tests, comparing models with linear terms against those incorporating spline terms [6].

Several sensitivity analyses were conducted to assess the robustness of the main findings: 1) Participants who died within the first 2 years of follow‐up were excluded to minimize reverse causality; 2) Given that chronic inflammation is a key pathophysiological mechanism linking body composition to mortality [18], we additionally adjusted for high‐sensitivity C‐reactive protein (hs‐CRP) to account for potential confounding by inflammatory status. Data on hsCRP were available for 1411 of the 2000 men (70.6%) and 1407 of the 2000 women (70.4%). To prevent exclusion of participants with missing data, we categorized hsCRP status based on the established cut‐off of 3 mg/L. [19, 20] Participants were accordingly classified into three groups: those with hsCRP ≤ 3 mg/L, those with hsCRP > 3 mg/L and those with missing hsCRP data; 3) To address the concern that low FLR might reflect subclinical cancer‐induced cachexia already present at baseline, we further excluded participants with incident cancer within the first 4 years of follow‐up to reduce possible reverse causality bias in cancer mortality analyses.

Finally, to determine whether FLR measures offer incremental predictive value over BMI, we compared their performance against BMI using the concordance index (c‐index) [21] derived from 10‐fold cross‐validation. Given that cause‐specific deaths were limited in number and that the train‐test split inherent to cross‐validation further reduces the sample size per fold, we pooled men and women to ensure adequate sample size for stable model fitting and reliable c‐index estimation.

A two‐sided *p*‐value of < 0.05 was considered statistically significant. All analyses were performed using R (version 4.1.0) and IBM SPSS (version 26.0) statistical software.

## 3 Results

### 3.1 Baseline Characteristics

This analysis included 2000 men (mean age: 72.4 ± 5.0 years) and 2000 women (mean age: 72.6 ± 5.4 years). The median follow‐up period was 18.2 years (IQR: 11.6–20.7 years). Table 1 presents the baseline characteristics stratified by whole‐body FLR tertiles. Age differed significantly across tertiles in both men (*p* = 0.002) and women (*p* = 0.011). In both sexes, participants in higher whole‐body FLR tertiles had significantly higher total and regional fat mass, as well as higher FLR values (all *p* < 0.001). With the exception of arm lean mass in women, total and regional absolute lean mass also differed significantly across the three groups (all *p* < 0.001). In men, higher FLR tertiles were associated with lower physical activity, higher prevalence of diabetes, hypertension, cardiovascular disease and greater use of antihypertensive and cholesterol‐lowering medications (all *p* < 0.05). Additionally, smoking status and caner prevalence also varied significantly across FLR groups in men (*p* < 0.05). In women, higher FLR tertiles were similarly associated with lower physical activity level, higher prevalence of hypertension and cardiovascular disease and greater use of antihypertensive medication (all *p* < 0.05). Diabetes prevalence also differed significantly across FLR tertiles in women (*p* < 0.01).

**TABLE 1:** Baseline characteristics for men and women by tertiles of whole‐body fat‐to‐lean mass ratio.

	Tertile 1 (lowest)	Tertile 2	Tertile 3 (highest)	*p*
Men (*n* = 2000, 50%)				
Age (years)	72.45 ± 5.15	71.88 ± 4.98	72.85 ± 4.85	0.002
Education				0.415
Illiterate	36 (5.4%)	37 (5.5%)	30 (4.5%)	
Primary or below	380 (57.1%)	370 (55.5%)	354 (53.1%)	
Secondary or above	250 (37.5%)	260 (39.0%)	283 (42.4%)	
Smoking				< 0.001
Non‐smoker	235 (35.3%)	243 (36.4%)	246 (36.9%)	
Ex‐smoker	325 (48.8%)	338 (50.7%)	375 (56.2%)	
Current smoker	106 (15.9%)	86 (12.9%)	46 (6.9%)	
Drinking (yes)	156 (23.4%)	175 (26.2%)	140 (21.0%)	0.078
PASE score	102.50 ± 53.54	99.94 ± 51.08	89.41 ± 45.01	< 0.001
Diabetes (yes)	75 (11.3%)	107 (16.0%)	111 (16.6%)	0.010
Hypertension (yes)	195 (29.3%)	285 (42.7%)	356 (53.4%)	< 0.001
Cardiovascular disease (yes)	125 (18.8%)	139 (20.8%)	188 (28.2%)	< 0.001
Cancer (yes)	38 (5.7%)	31 (4.6%)	18 (2.7%)	0.024
Antihypertensive medication (yes)	226 (33.9%)	298 (44.7%)	374 (56.1%)	< 0.001
Cholesterol lowering medication (yes)	34 (5.1%)	46 (6.9%)	72 (10.8%)	< 0.001
Total lean mass (kg)	42.66 ± 5.60	44.91 ± 5.21	45.13 ± 5.24	< 0.001
Trunk lean mass (kg)	20.80 ± 2.84	21.94 ± 2.63	22.20 ± 2.73	< 0.001
Abdominal lean mass (kg)	5.42 ± 0.86	5.90 ± 0.83	6.21 ± 0.92	< 0.001
Arm lean mass (kg)	4.87 ± 0.75	5.10 ± 0.74	5.03 ± 0.70	< 0.001
Leg lean mass (kg)	13.70 ± 2.02	14.44 ± 1.92	14.38 ± 1.90	< 0.001
Total fat mass (kg)	10.59 ± 2.90	15.51 ± 2.04	19.78 ± 3.20	< 0.001
Trunk fat mass (kg)	5.66 ± 1.99	8.89 ± 1.48	11.49 ± 2.10	< 0.001
Abdominal fat mass (kg)	1.47 ± 0.60	2.42 ± 0.48	3.26 ± 0.71	< 0.001
Arm fat mass (kg)	1.14 ± 0.36	1.69 ± 0.31	2.19 ± 0.45	< 0.001
Leg fat mass (kg)	2.93 ± 0.79	4.03 ± 0.77	5.18 ± 1.10	< 0.001
Whole‐body FLR	0.25 ± 0.05	0.35 ± 0.02	0.44 ± 0.05	< 0.001
Trunk FLR	0.27 ± 0.08	0.40 ± 0.04	0.52 ± 0.07	< 0.001
Abdominal FLR	0.26 ± 0.09	0.41 ± 0.05	0.52 ± 0.08	< 0.001
Arm FLR	0.23 ± 0.06	0.33 ± 0.05	0.44 ± 0.08	< 0.001
Leg FLR	0.21 ± 0.05	0.28 ± 0.05	0.36 ± 0.07	< 0.001
Women (*n* = 2000, 50%)				
Age (years)	73.08 ± 5.95	72.25 ± 5.10	72.41 ± 4.95	0.011
Education				0.394
Illiterate	266 (39.9%)	242 (36.3%)	245 (36.8%)	
Primary or below	290 (43.5%)	299 (44.8%)	314 (47.1%)	
Secondary or above	111 (16.6%)	126 (18.9%)	107 (16.1%)	
Smoking				0.280
Non‐smoker	591 (88.6%)	609 (91.3%)	610 (91.6%)	
Ex‐smoker	59 (8.8%)	47 (7.0%)	47 (7.1%)	
Current smoker	17 (2.5%)	11 (1.6%)	9 (1.4%)	
Drinking (yes)	15 (2.2%)	15 (2.2%)	21 (3.2%)	0.481
PASE score	87.41 ± 34.17	86.59 ± 31.43	82.09 ± 33.62	0.007
Diabetes (yes)	116 (17.4%)	95 (14.2%)	75 (11.3%)	0.006
Hypertension (yes)	237 (35.5%)	308 (46.2%)	326 (48.9%)	< 0.001
Cardiovascular disease (yes)	103 (15.4%)	127 (19.0%)	150 (22.5%)	0.004
Cancer (yes)	35 (5.2%)	26 (3.9%)	29 (4.4%)	0.481
Antihypertensive medication (yes)	227 (34.0%)	297 (44.5%)	320 (48.0%)	< 0.001
Cholesterol lowering medication (yes)	62 (9.3%)	61 (9.1%)	85 (12.8%)	0.050
Total lean mass (kg)	32.84 ± 4.07	33.99 ± 3.92	34.67 ± 4.11	< 0.001
Trunk lean mass (kg)	16.48 ± 2.14	17.12 ± 2.12	17.52 ± 2.17	< 0.001
Abdominal lean mass (kg)	4.63 ± 0.68	4.97 ± 0.67	5.27 ± 0.73	< 0.001
Arm lean mass (kg)	3.41 ± 0.52	3.46 ± 0.50	3.46 ± 0.54	0.109
Leg lean mass (kg)	10.07 ± 1.47	10.41 ± 1.40	10.63 ± 1.48	< 0.001
Total fat mass (kg)	14.05 ± 3.20	19.06 ± 2.41	24.20 ± 3.77	< 0.001
Trunk fat mass (kg)	7.18 ± 2.08	10.01 ± 1.60	12.60 ± 2.15	< 0.001
Abdominal fat mass (kg)	1.88 ± 0.60	2.73 ± 0.51	3.55 ± 0.72	< 0.001
Arm fat mass (kg)	1.78 ± 0.54	2.48 ± 0.47	3.27 ± 0.71	< 0.001
Leg fat mass (kg)	4.29 ± 1.15	5.75 ± 1.17	7.48 ± 1.66	< 0.001
Whole‐body FLR	0.43 ± 0.07	0.56 ± 0.03	0.70 ± 0.07	< 0.001
Trunk FLR	0.43 ± 0.10	0.58 ± 0.06	0.72 ± 0.09	< 0.001
Abdominal FLR	0.40 ± 0.10	0.55 ± 0.07	0.67 ± 0.09	< 0.001
Arm FLR	0.52 ± 0.14	0.72 ± 0.11	0.95 ± 0.18	< 0.001
Leg FLR	0.43 ± 0.10	0.55 ± 0.10	0.71 ± 0.13	< 0.001

*Note:* FLR, fat‐to‐lean mass ratio; PASE, Physical Activity Scale for the Elderly. Continuous variables were presented as mean (SD) and compared using one‐way analysis of variance (ANOVA). Categorical variables were presented as number (%) and was compared using chi‐square test. The sample sizes of Tertile 1, Tertile 2 and Tertile 3 in men were 666, 667 and 667, respectively. The sample sizes of Tertile 1, Tertile 2 and Tertile 3 in women were 667, 667 and 666, respectively.

### 3.2 Fat‐to‐Lean Mass Ratio and Mortality

**FIGURE 2:**
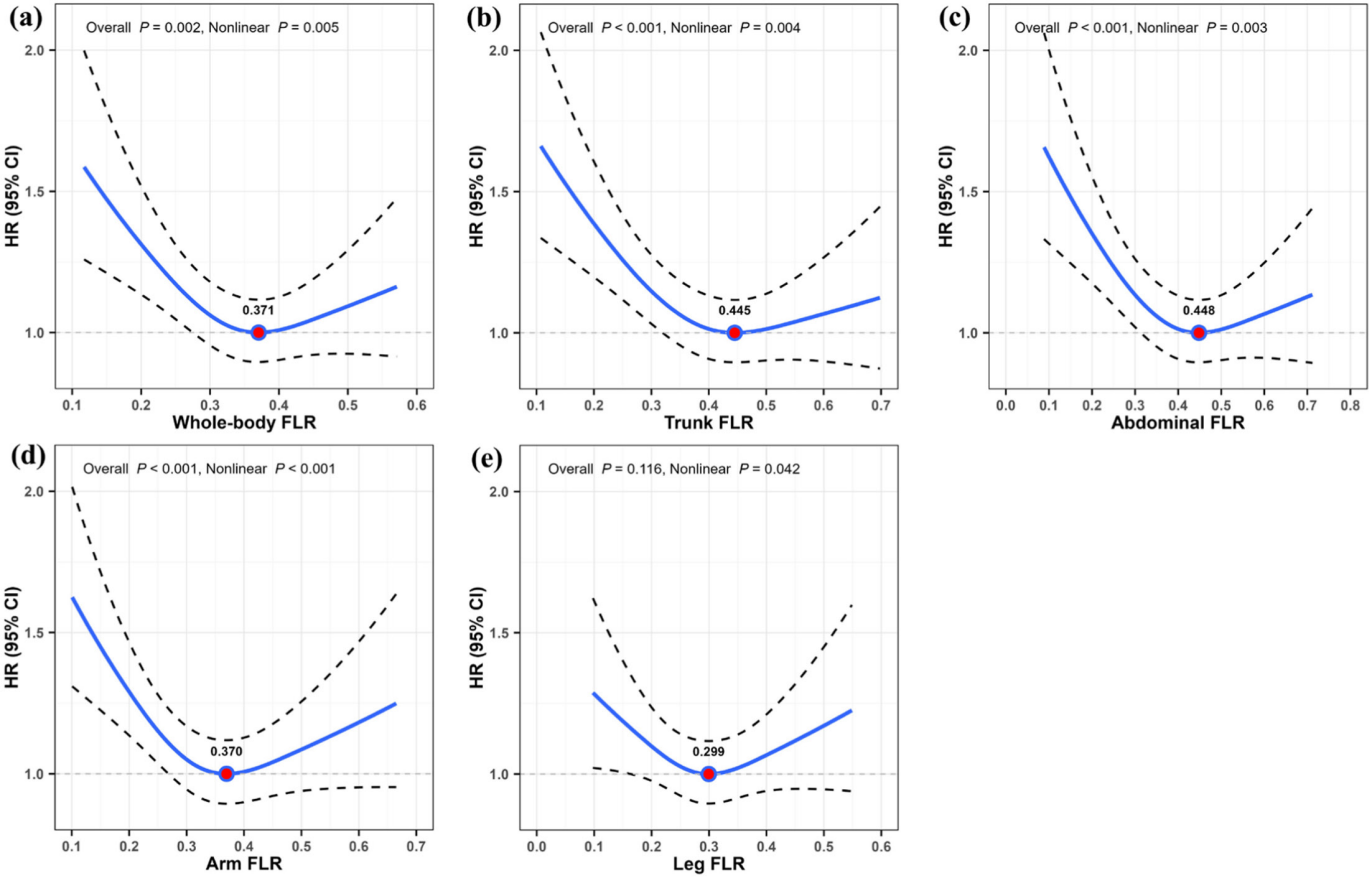
The associations of FLR with all‐cause mortality for men. (a) for whole‐body, (b) for trunk, (c) for abdominal, (d) for arm and (e) for leg. All models were adjusted for age, educational level, smoking status, drinking, physical activity, diabetes, hypertension, cardiovascular disease, cancer, antihypertensive medication, cholesterol lowering medication and total or regional absolute lean mass. Hazard ratios are reported by solid lines and 95% CIs by dashed areas. The reference point is the FLR at minimum mortality risk, using three knots at the 10th, 50th and 90th percentiles. Abbreviations: aHR: adjusted hazard ratio; CI: confidence interval; FLR: Fat‐to‐lean mass ratio.

**FIGURE 3:**
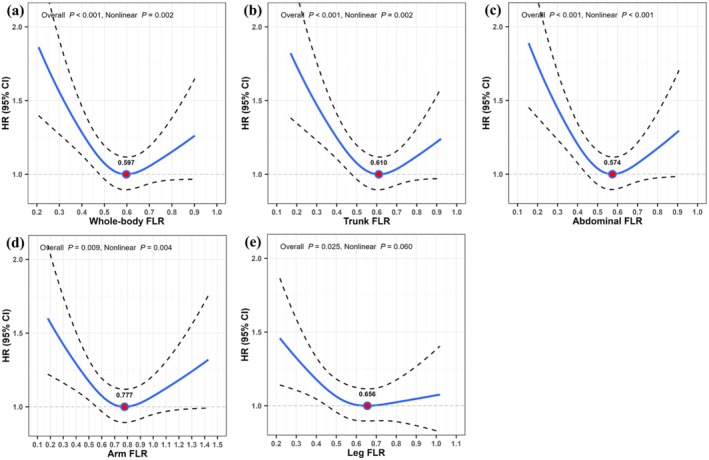
The associations of FLR with all‐cause mortality for women. (a) for whole‐body, (b) for trunk, (c) for abdominal, (d) for arm and (e) for leg. All models were adjusted for age, educational level, smoking status, drinking, physical activity, diabetes, hypertension, cardiovascular disease, cancer, antihypertensive medication, cholesterol lowering medication and total or regional absolute lean mass. Hazard ratios are reported by solid lines and 95% CIs by dashed areas. The reference point is the FLR at minimum mortality risk, using three knots at the 10th, 50th and 90th percentiles. Abbreviations: aHR: adjusted hazard ratio; CI: confidence interval; FLR: Fat‐to‐lean mass ratio.

The 1‐, 5‐, 10‐, 15‐ and 20‐year survival rates were 0.990, 0.897, 0.753, 0.557 and 0.354 for men, and 0997, 0.960, 0.858, 0.708 and 0.500 for women (Table S1). Univariate survival analyses by Kaplan–Meier curves (Figure S1 and Figure S2) for all‐cause mortality revealed that both total and regional FLRs were statistically associated with mortality, except for trunk and arm FLRs in women. The multivariable‐adjusted associations between FLR and all‐cause mortality are shown in Table 2. In men, FLR was inversely associated with mortality risk when analysed as continuous form. Specifically, each SD increase in the whole‐body FLR was associated with a 6% reduction in mortality risk (HR: 0.94, 95% CI: 0.89–1.00, *p* = 0.035). This inverse association was also significant for the trunk (HR: 0.92, 95% CI: 0.87–0.97, *p* = 0.003) and abdominal region (HR: 0.92, 95% CI: 0.86–0.97, *p* = 0.004), as well as for the arms (HR: 0.94, 95% CI: 0.89–1.00, *p* = 0.037). However, no significant association was observed for the leg FLR (*p* = 0.606). When examining FLR by tertiles in men, those in the lowest tertile (T1) of whole‐body FLR had a significantly higher risk of mortality compared to the T2, with a 20% increased risk (HR: 1.20, 95% CI: 1.05–1.36, *p* = 0.008). A similar pattern was observed for regional measures, where the lowest tertile of FLR in the trunk (HR: 1.26, 95% CI: 1.10–1.44, *p* = 0.001), abdominal (HR: 1.18, 95% CI: 1.03–1.35, *p* = 0.017) and arms (HR: 1.25, 95% CI: 1.10–1.43, *p* = 0.001) was associated with a significantly higher mortality risk. No significant associations were found for the highest tertile (T3) in any region in men. In women, the continuous analysis revealed that FLR was also inversely associated with mortality risk for the trunk (HR per SD increase: 0.94, 95% CI: 0.88–1.00, *p* = 0.041) and abdominal region (HR: 0.91, 95% CI: 0.85–0.97, *p* = 0.007). The inverse associations for whole‐body FLR (HR: 0.94, 95% CI: 0.88–1.00, *p* = 0.051) and leg FLR (HR: 0.94, 95% CI: 0.88–1.00, *p* = 0.053) were of a similar magnitude but did not reach statistical significance. In the categorical analysis, women in the lowest tertile of abdominal FLR had an 18% higher risk of mortality compared to the T2 (HR: 1.18, 95% CI: 1.01–1.37, *p* = 0.036). No other significant associations were found across the tertiles for any other body region in women. The restricted cubic spline analyses revealed L‐shaped associations of total and regional FLR with all‐cause mortality (Figure 2 and Figure 3). These non‐linear relationships were statistically significant for all FLR measures in both sexes (*p* for non‐linearity: < 0.05), except a borderline significance for leg FLR in women (*p* for non‐linearity: 0.060).

**TABLE 2:** Multivariable‐adjusted HRs (95% CIs) for all‐cause mortality by total and regional fat‐to‐lean ratio using Cox regression model.

	Men		Women	
	HR (95% CI)	*p*	HR (95% CI)	*p*
Whole body				
Continuous	0.94 (0.89–1.00)	0.035	0.94 (0.88–1.00)	0.051
Tertile 1	1.20 (1.05–1.36)	0.008	1.16 (0.99–1.35)	0.062
Tertile 2	1 (reference)	—	1 (reference)	—
Tertile 3	1.10 (0.96–1.26)	0.157	1.10 (0.95–1.29)	0.177
Trunk				
Continuous	0.92 (0.87–0.97)	0.003	0.94 (0.88–1.00)	0.041
Tertile 1	1.26 (1.10–1.44)	0.001	1.13 (0.98–1.32)	0.104
Tertile 2	1 (reference)	—	1 (reference)	—
Tertile 3	1.08 (0.94–1.23)	0.270	1.03 (0.88–1.19)	0.735
Abdominal				
Continuous	0.92 (0.86–0.97)	0.004	0.91 (0.85–0.97)	0.007
Tertile 1	1.18 (1.03–1.35)	0.017	1.18 (1.01–1.37)	0.036
Tertile 2	1 (reference)	—	1 (reference)	—
Tertile 3	1.02 (0.90–1.17)	0.721	0.99 (0.85–1.16)	0.916
Arm				
Continuous	0.94 (0.89–1.00)	0.037	0.96 (0.91–1.03)	0.238
Tertile 1	1.25 (1.10–1.43)	0.001	1.03 (0.89–1.20)	0.662
Tertile 2	1 (reference)	—	1 (reference)	—
Tertile 3	1.12 (0.98–1.28)	0.093	1.02 (0.88–1.19)	0.782
Leg				
Continuous	0.99 (0.93–1.04)	0.606	0.94 (0.88–1.00)	0.053
Tertile 1	1.13 (0.99–1.28)	0.077	1.16 (0.99–1.35)	0.061
Tertile 2	1 (reference)	—	1 (reference)	—
Tertile 3	1.13 (0.99–1.29)	0.064	1.02 (0.88–1.19)	0.782

*Note:* All models were adjusted for age, educational level, smoking status, drinking, physical activity, diabetes, hypertension, cardiovascular disease, cancer, antihypertensive medication, cholesterol lowering medication and the whole‐body or regional absolute lean mass. Results were reported by HR and 95% CI. For continuous FLR, HR per one standard deviation increase for each FLR indicator was used.

Abbreviations: CI, confidence interval; FLR, fat‐to‐lean mass ratio; HR, hazard ratio.

For cause‐specific mortality (Table 3), no statistically significant associations were observed for CVD mortality in either men or women. For cancer mortality, a lower leg FLR was significantly associated with a higher risk only in men. This association was significant in both continuous (HR per SD increase: 0.89; 95% CI: 0.80–0.99; *p* = 0.036) and categorical analyses, where the lowest tertile (T1) showed an increased risk (HR: 1.29; 95% CI: 1.00–1.66; *p* = 0.047) compared to T2. In women, no significant associations with cancer mortality were observed for most FLR measures (analysed either continuously or categorically), except for the trunk FLR (HR per‐SD increase: 1.17; 95% CI: 1.00–1.35; *p* = 0.047).

**TABLE 3:** Multivariable‐adjusted HRs (95% CIs) for cause‐specific mortality by total and regional fat‐to‐lean ratio using Fine–Gray competing risk model.

	CVD‐cause mortality				Cancer‐cause mortality			
	Men		Women		Men		Women	
	HR (95% CI)	*p*	HR (95% CI)	*p*	HR (95% CI)	*p*	HR (95% CI)	*p*
Whole body								
Continuous	1.06 (0.92–1.23)	0.432	0.90 (0.76–1.07)	0.244	0.92 (0.82–1.03)	0.138	1.15 (0.99–1.33)	0.065
Tertile 1	0.91 (0.64–1.30)	0.599	1.38 (0.95–2.02)	0.095	1.03 (0.80–1.33)	0.808	1.02 (0.73–1.42)	0.927
Tertile 2	1 (reference)	—	1 (reference)	—	1 (reference)	—	1 (reference)	—
Tertile 3	1.16 (0.83–1.62)	0.391	1.27 (0.86–1.87)	0.228	0.93 (0.72–1.21)	0.609	1.18 (0.86–1.62)	0.319
Trunk								
Continuous	1.04 (0.89–1.22)	0.604	0.89 (0.75–1.05)	0.176	0.94 (0.84–1.05)	0.265	1.17 (1.00–1.35)	0.047
Tertile 1	1.01 (0.71–1.44)	0.962	1.32 (0.92–1.91)	0.135	1.20 (0.93–1.54)	0.169	0.91 (0.65–1.27)	0.573
Tertile 2	1 (reference)	—	1 (reference)	—	1 (reference)	—	1 (reference)	—
Tertile 3	1.13 (0.80–1.59)	0.483	1.03 (0.69–1.52)	0.898	1.12 (0.86–1.45)	0.416	1.24 (0.90–1.69)	0.183
Abdominal								
Continuous	1.03 (0.88–1.20)	0.760	0.88 (0.74–1.05)	0.167	0.96 (0.86–1.08)	0.520	1.08 (0.92–1.27)	0.322
Tertile 1	0.82 (0.57–1.18)	0.284	1.34 (0.92–1.94)	0.128	1.14 (0.88–1.47)	0.337	0.93 (0.66–1.29)	0.648
Tertile 2	1 (reference)	—	1 (reference)	—	1 (reference)	—	1 (reference)	—
Tertile 3	0.91 (0.65–1.28)	0.598	1.07 (0.72–1.58)	0.748	1.09 (0.84–1.41)	0.536	1.01 (0.73–1.38)	0.975
Arm								
Continuous	1.07 (0.93–1.24)	0.347	0.92 (0.78–1.09)	0.344	0.92 (0.82–1.03)	0.136	1.14 (0.99–1.31)	0.077
Tertile 1	1.01 (0.71–1.43)	0.973	1.15 (0.80–1.66)	0.441	1.21 (0.94–1.56)	0.142	0.89 (0.64–1.24)	0.488
Tertile 2	1 (reference)	—	1 (reference)	—	1 (reference)	—	1 (reference)	—
Tertile 3	1.19 (0.84–1.68)	0.329	1.03 (0.69–1.52)	0.896	1.13 (0.86–1.47)	0.383	1.12 (0.82–1.54)	0.470
Leg								
Continuous	1.10 (0.97–1.25)	0.154	0.96 (0.82–1.13)	0.627	0.89 (0.80–0.99)	0.036	1.06 (0.93–1.21)	0.395
Tertile 1	0.84 (0.59–1.19)	0.315	1.25 (0.86–1.83)	0.249	1.29 (1.00–1.66)	0.047	1.10 (0.79–1.52)	0.580
Tertile 2	1 (reference)	—	1 (reference)	—	1 (reference)	—	1 (reference)	—
Tertile 3	1.09 (0.78–1.53)	0.618	1.23 (0.84–1.81)	0.292	1.09 (0.84–1.42)	0.527	1.12 (0.81–1.55)	0.497

*Note:* All models were adjusted for age, educational level, smoking status, drinking, physical activity, diabetes, hypertension, cardiovascular disease (for cancer‐cause), cancer (for CVD‐cause), antihypertensive medication, cholesterol lowering medications and the whole‐body or regional absolute lean mass. Results were reported by HR and 95% CI. For continuous FLR (fat‐to‐lean mass ratio), HR per one standard deviation increase was used. HR, hazard ratio; CI, confidence interval. For CVD‐cause analysis: in men, the analytical sample was 1541 (seven cases without death causes and 452 participants with baseline CVD were excluded); in women, the analytical sample size was 1618 (two cases without death causes and 380 participants with baseline CVD were excluded). For Cancer‐cause analysis: in men, the analytical sample was 1907 (seven cases without death causes and 86 participants with baseline cancer were excluded); in women, the analytical sample size was 1909 (two cases without death causes and 89 participants with baseline cancer were excluded).

### 3.3 Sensitivity Analyses

All sensitivity analyses, including exclusion of early deaths (Table S2 and Table S3), adjustment for hsCRP (Table S4 and Table S5) and exclusion of early cancer incidents (Table S6), confirmed the robustness of our main findings. Of note, the association between trunk FLR and cancer mortality in women was no longer statistically significant in all sensitivity analyses.

### 3.4 Discriminative Ability of FLR and BMI in Estimating Mortality Risk

The predictive performance of different body composition measures for mortality, assessed by the c‐index derived from 10‐fold cross‐validation, is presented in Table 4. For all‐cause mortality, the c‐indices of FLR measures ranged from 0.554 to 0.564, which were significantly higher than the 0.522 observed for BMI (all *p* < 0.001). Similar patterns were observed for CVD mortality (c‐indices of FLRs: 0.535–0.546 vs. 0.474 for BMI, all *p* < 0.01) and cancer mortality (c‐indices of FLRs: 0.552–0.576 vs. 0.477 for BMI, all *p* < 0.001).

**TABLE 4:** Predictive performance (measured using c‐index) between different body composition measures in estimating mortality risk.

	All‐cause		CVD‐cause		Cancer‐cause	
	C‐index	Difference	C‐index	Difference	C‐index	Difference
BMI	0.522 ± 0.014	Reference	0.474 ± 0.023	Reference	0.477 ± 0.028	Reference
Whole‐body FLR	0.564 ± 0.016	0.042***	0.545 ± 0.041	0.071***	0.572 ± 0.034	0.095***
Trunk FLR	0.555 ± 0.013	0.033***	0.537 ± 0.041	0.063**	0.560 ± 0.032	0.083***
Abdominal FLR	0.554 ± 0.016	0.032***	0.535 ± 0.046	0.061**	0.552 ± 0.026	0.075***
Arm FLR	0.562 ± 0.016	0.040***	0.546 ± 0.036	0.072***	0.573 ± 0.034	0.096***
Leg FLR	0.561 ± 0.020	0.039***	0.545 ± 0.044	0.071***	0.576 ± 0.030	0.099***

*Note:* Results in the above table were presented as mean and standard deviation through 10‐fold cross validation. FLR, fat‐to‐lean mass ratio. *, ** and *** represent *P* < 0.05, *P* < 0.01 and *p* < 0.001, respectively. Statistical comparisons were performed between BMI and FLR indicators (BMI used as reference, difference in c‐index was calculated by each FLR indicator minus BMI).

## 4 Discussion

### 4.1 Main Findings

In this prospective cohort of community‐dwelling Chinese older adults followed for up to 20 years, we observed that lower total and regional fat‐to‐lean mass ratio was associated with higher all‐cause mortality in both sexes, particularly in men. These associations exhibited L‐shaped non‐linear patterns, with the excess mortality risk largely confined to the lowest FLR tertile. For cause‐specific mortality, no significant associations were observed for CVD mortality in either sex. However, a lower leg FLR was associated with increased cancer mortality only in men, an association that remained robust after excluding early cancer incidents. These findings underscore the importance of evaluating both the absolute amount and the anatomical distribution of fat and lean mass when assessing mortality risk in older adults.

### 4.2 Comparisons With Previous Studies Regarding FLR and All‐Cause Mortality

It is challenging to directly compare our findings with existing literature due to substantial differences in population characteristics, methodology and definitions of body composition. Most prior studies were conducted in western populations using BIA to examine the relationships between FMR and mortality. In contrast, our study focused on an older Chinese cohort and utilized DXA to investigate the associations between FLR and mortality. Despite these differences, the inverse association between FLR and all‐cause mortality in our study aligns with some existing findings. For example, Xu et al. reported that individuals in quintiles 2–4 of whole‐body and trunk FMR had a 12%–14% and 7%–15% lower all‐cause mortality risk, respectively, compared to those in the lowest quintile. Moreover, the hazard ratios for leg FMR comparing the highest to the lowest quintiles were 0.76 (95% CI: 0.71–0.82) in men and 0.78 (95% CI: 0.70–0.88) in women [10]. Our study further demonstrated that these inverse associations remain significant after adjusting for absolute lean mass. Meanwhile, our RCS analyses suggest that the minimum all‐cause mortality risk occurs around the middle tertile of FLR distributions, with approximate risk nadirs of 0.37 in men and 0.60 in women for whole‐body FLR. Whereas establishing definitive clinical cut‐off points is beyond the scope of this single study. Such thresholds would require further validation in large and diverse populations.

The mechanisms underlying the elevated mortality risk associated with a low FLR in older adults may be multifactorial. Importantly, this phenomenon may be reinterpreted within the context of the ‘obesity paradox’, where the survival disadvantage of low FLR in older age may be driven by unintentional weight loss due to subclinical chronic diseases, systemic inflammation, or pre‐frailty [22, 23]. Thus, the observed higher mortality in the lowest FLR group may reflect a state of vulnerability, characterized by depleted energy and protein reserves. In contrast, higher FLR groups likely represent those with maintained anabolic homeostasis. Our analysis showed that individuals with high FLR values generally had both higher absolute lean and fat mass (shown in Table 1). The preservation of lean or muscle mass likely supports physical function and metabolic fitness [24], whereas the accompanying fat may provide energy stores during periods of acute illness or metabolic stress [25]. For example, the gluteofemoral fat mass, often termed “beneficial fat”, has been independently associated with a favourable lipid and glucose profile and a reduced risk of cardiovascular and metabolic diseases [26], possibly through the secretion of adiponectin, which enhances metabolic homeostasis and exerts anti‐inflammatory effects [27]. However, we caution against extrapolating this to trunk or abdominal FLR, as DXA cannot distinguish between the potentially neutral or protective subcutaneous fat and pathogenic visceral adipose tissue. Additionally, in advanced age, a higher FLR may also reflect overall nutritional adequacy, countering the risks associated with weight loss and sarcopenia, which are potent predictors of mortality [28]. Moreover, our sensitivity analyses adjusting for high‐sensitivity C‐reactive protein (hs‐CRP) did not materially change the estimates, suggesting that the association of low FLR with higher mortality may not fully explained by systemic inflammation. Although we did observe weak but significantly positive correlations between hs‐CRP and some FLR measures in men (*r* = 0.069 for total FLR, *r* = 0.074 for trunk FLR) and women (*r* = 0.046–0.078 for most regions except leg), these small values indicate that the survival disadvantage for low FLR people may operate through pathways beyond conventional inflammatory mechanisms. Due to lack of additional inflammation data, this explanation requires further validation.

### 4.3 Null Associations Between FLR and Cardiovascular Mortality

We found no significant associations between any FLR measure and CVD mortality in either sex. This null finding in women aligns with a previous UK Biobank study which also reported no significant association between fat‐to‐muscle mass ratio and CVD mortality in women [8]. Although they observed significant positive associations for trunk and arm fat‐to‐muscle mass ratio with CVD mortality in men [8], this pattern was not replicated in our older male cohort. This sex‐specific discrepancy may be primarily attributed to the differences in population characteristics. First, our cohort comprised older adults aged ≥ 65 years (mean age: 72 years), whereas the UK Biobank study included predominantly middle‐aged participants (mean age: 56 years). In older populations, the relationship between body composition and CVD mortality may attenuate, as competing risks from non‐CVD causes (e.g., frailty, infections, cancer) become increasingly dominant [29]. Second, ethnic differences between Chinese and western populations may also contribute, given well‐documented variations in body composition and fat distribution across ethnic groups [30, 31].

### 4.4 Sex‐Specific Association of FLR With Cancer Mortality

Interestingly, we found that a lower leg FLR was associated with higher cancer mortality in men, which remained robust after excluding participants with incident cancer within the first 4 years of follow‐up. In light of this finding, several possible explanations may be considered. First, leg fat, particularly gluteofemoral fat, acts as a beneficial component and is independently associated with favourable metabolic parameters and elevated adiponectin levels. Adiponectin further helps maintain metabolic homeostasis and exerts anti‐inflammatory effects, thereby potentially reducing cancer risk [26, 27]. Thus, men with lower leg FLR may have reduced leg fat mass and consequently lower adiponectin levels, which could partially explain their increased cancer mortality risk. Second, lower leg FLR may also reflect reduced leg muscle mass. Leg muscle mass, being the largest muscle depot in the body, plays a critical role in glucose homeostasis and insulin sensitivity [32]. Reduced leg muscle mass may impair insulin sensitivity, leading to higher circulating levels of insulin and insulin‐like growth factors, which promote cell proliferation and inhibit apoptosis, thereby potentially increasing cancer risk [32]. However, given that DXA‐derived FLR cannot disentangle the relative contributions of fat versus lean mass to the ratio, the above interpretation remains speculative. Besides, the sex‐specific nature of this association may be partially explained by hormonal differences. In men, we observed significant negative correlations between testosterone and all FLR measures (correlation coefficients ranging from −0.224 to −0.342, all *p* < 0.001), with these associations remaining robust even after adjusting for absolute lean mass (partial correlation coefficients ranging from −0.234 to −0.336, all *p* < 0.001). This suggests that men with lower FLR have higher testosterone levels, independent of their lean mass. Given that elevated testosterone can promote the development of certain cancers (e.g., prostate cancer, malignant melanoma, liver cancer) [33, 34], this hormonally‐mediated pathway may partly account for the observed increased cancer mortality risk among men with lower leg FLR. In women, we observed a borderline positive association between trunk FLR and cancer mortality, but was not significant after adjusting hsCRP. This attenuation upon adjustment is consistent with our correlation data showing that, in women, trunk and abdominal fat mass were significantly correlated with hsCRP (correlation coefficients of 0.081 and 0.075, respectively, both *p* < 0.01), whereas leg fat mass showed no significant correlation. These findings suggest that the pro‐inflammatory nature of trunk or abdominal fat, which includes a substantial proportion of visceral adipose tissue (VAT), may underlie the adverse cancer mortality trend observed in women. Although DXA cannot directly quantify VAT, our abdominal FLR measure likely captures some component of visceral adiposity. Considering that the association between lower leg FLR and higher cancer mortality was observed only in men and not in other regions or in women, this finding should be interpreted with caution and warrants further validation. Moreover, given the absence of further data on blood biomarkers (e.g., oestrogen) and genetic information in our cohort, this study cannot uncover the exact mechanisms underlying the above findings, we only propose these potential hypotheses for validation in future research.

### 4.5 Discriminative Ability of FLR and BMI in Mortality Risk Stratification

In terms of predictive performance, FLR measures demonstrated higher predictive performance than BMI in estimating both all‐cause and cause‐specific mortality, with c‐indices ranging from 0.535 to 0.576 for FLRs compared to 0.474 to 0.522 for BMI (all *p* < 0.01). However, we acknowledge that these absolute c‐index values indicate relatively low discriminatory power. This is not unexpected given the multifactorial nature of mortality risk in older adults, where body composition represents just one of many determinants. The clinical utility of FLR may therefore lie not in standalone risk prediction but in its ability to provide incremental information beyond BMI. Future studies integrating FLR with comprehensive geriatric assessments and other biomarkers may achieve improved predictive performance.

### 4.6 Strengths, Implications and Limitations

This study has several key strengths, including a long follow‐up period, the use of DXA to assess body composition, sex‐stratified analyses on both all‐cause and cause‐specific mortality, comprehensive adjustment for potential confounders such as absolute lean mass and inflammatory markers and rigorous sensitivity analyses to rule out reverse causality. However, several limitations of this study should be considered. First, the analysis was conducted exclusively among older Chinese adults, which may limit the generalizability of our findings to other ethnicities or younger populations. Additionally, the number of cause‐specific deaths was relatively modest, which limited our statistical power for subgroup analyses. Future multi‐ethnic and larger‐scale studies are warranted to validate these findings. Second, although we adjusted for several confounders, residual confounding cannot be fully excluded, such as other inflammatory factors (e.g., interleukin‐6, tumour necrosis factor‐α), hormones (e.g., oestrogen), environmental exposures and genetic predispositions [35]. Future research incorporating a broader panel of this information would help clarify the mechanisms. Third, although DXA is a precise tool for assessing body composition, a key limitation is that DXA only measures total lean mass and cannot isolate skeletal muscle from other components, such as body water and connective tissue. This limitation is particularly relevant for trunk FLR, where the lean compartment includes vital organs rather than just muscle. Furthermore, DXA cannot distinguish between subcutaneous adipose tissue, visceral adipose tissue and intramuscular or intermuscular adipose tissue (i.e., myosteatosis). This requires particular attention when interpreting our findings in trunk and abdominal regions, as VAT is highly pathogenic and pro‐inflammatory [36], whereas subcutaneous fat may be metabolically neutral or beneficial [36] and myosteatosis is associated with impaired physical function and increased mortality [37]. Therefore, future studies employing more precise imaging techniques such as computed tomography (CT) or magnetic resonance imaging (MRI) are needed to further quantify muscle quality, differentiate between visceral and subcutaneous fat and clarify the relationships of specific fat depots with mortality. Fourth, we acknowledge that, within the high FLR group, a certain proportion of individuals may have sarcopenic obesity or myosteatosis, which would be associated with higher mortality risk [37, 38]. However, in our analysis, the high FLR participants generally had significantly higher lean mass, as well as higher fat mass, compared to those with low FLR (Table 1). This suggests that, even if some individuals had impaired muscle quality, the overall survival advantage observed in the high FLR group may still be driven, at least in part, by preserved anabolic reserve. Nonetheless, future studies using CT or MRI are needed to directly quantify myosteatosis and clarify the relationships of muscle quality with mortality. Fifth, body composition was only assessed at baseline in our study and changes over the 20‐year follow‐up period were not captured. Longitudinal assessments with repeated DXA measurements would provide valuable insights into how changes in FLR over time relate to mortality risk. Finally, although we excluded early deaths and early caner incidents in sensitivity analyses, residual reverse causality cannot be completely ruled out. For instance, individuals with undiagnosed chronic diseases, subclinical cachexia, or pre‐frailty at baseline may have already experienced unintentional weight loss and muscle wasting, leading to a low FLR and subsequently higher mortality risk. In contrast, the group with higher FLR may simply represent individuals who still maintain their anabolic homeostasis intact.

## 5 Conclusions

In summary, this study demonstrates that a lower fat‐to‐lean mass ratio is associated with higher all‐cause mortality in Chinese older adults of both sexes, exhibiting L‐shaped relationships. No significant associations were observed for CVD mortality and a lower leg FLR was associated with higher cancer mortality in men, but not in women. The DXA‐derived FLRs may serve as useful risk stratification indicators to identify older adults at higher risk of mortality due to the depletion of energetic and muscular reserves.

## Author Contributions

Yafei Wu: Conceptualization, Methodology, Formal analysis, Visualization, Writing – original draft, Writing‐review and editing. Ting Zhang: Methodology, Writing – review and editing. Shuyi Li: Methodology. Jason Leung: Data curation. Timothy Kwok: Conceptualization, Funding acquisition, Supervision, Writing – review and editing.

## Funding

This study was supported by the National Institutes of Health R01 grant (grant number: AR049439–01A1) and the Research Grants Council Earmarked Grant (grant number: CUHK4101/02 M).

## Ethics Statement

The study was carried out in accordance with the ethical standards laid down in the 1964 Declaration of Helsinki and its later amendments and was approved by the Clinical Research Ethics Committee of The Chinese University of Hong Kong. All participants provided informed consent. All authors certify that they comply with the Ethical guidelines for authorship and publishing in the Journal of Cachexia, Sarcopenia and Muscle.

## Conflicts of Interest

The authors declare no conflicts of interest.

## Supporting information


**Table S1:** The survival rate of study sample in the Mr. OS & Ms. OS (Hong Kong) cohort.
**Table S2:** Multivariable‐adjusted HRs (95% CIs) for all‐cause mortality by total and regional FLR with excluding those who died within 2 years after baseline survey using Cox regression model.
**Table S3:** Multivariable‐adjusted HRs (95% CIs) for cause‐specific mortality by total and regional FLR with excluding those who died within 2 years after baseline survey using Fine–Gray competing risk model.
**Table S4:** Multivariable‐adjusted HRs (95% CIs) for all‐cause mortality by total and regional fat‐to‐lean ratio using Cox regression model (adjusting hsCRP).
**Table S5:** Multivariable‐adjusted HRs (95% CIs) for cause‐specific mortality by total and regional fat‐to‐lean ratio using Fine–Gray competing risk model (adjusting hsCRPP).
**Table S6:** Multivariable‐adjusted HRs (95% CIs) for cancer‐specific mortality by total and regional fat‐to‐lean ratio using Fine–Gray competing risk model (excluding those who had incident cancer within the first 4‐year follow up).
**Figure S1:** The Kaplan–Meier curves of all‐cause mortality stratified by total and regional fat‐to‐lean mass ratio levels in men.
**Figure S2:** The Kaplan–Meier curves of all‐cause mortality stratified by total and regional fat‐to‐lean mass ratio levels in women.

## Data Availability

The data that support the findings of this study are available from the corresponding author upon reasonable request.

## References

[1] Y. C. Wang , K. McPherson , T. Marsh , S. L. Gortmaker , and M. Brown , “Health and Economic Burden of the Projected Obesity Trends in the USA and the UK,” Lancet 378 (2011): 815–825. 21872750 10.1016/S0140-6736(11)60814-3

[2] F. X. Pi‐Sunyer , “The Medical Risks of Obesity,” Obesity Surgery 12 (2002): 6s–11s. 11969107 10.1007/BF03342140

[3] A. Romero‐Corral , V. K. Somers , J. Sierra‐Johnson , et al., “Accuracy of Body Mass Index in Diagnosing Obesity in the Adult General Population,” International Journal of Obesity 32 (2008): 959–966. 18283284 10.1038/ijo.2008.11 PMC2877506

[4] A. Bosy‐Westphal , W. Braun , C. Geisler , K. Norman , and M. J. Müller , “Body Composition and Cardiometabolic Health: The Need for Novel Concepts,” European Journal of Clinical Nutrition 72 (2018): 638–644. 29748654 10.1038/s41430-018-0158-2

[5] P. C. Yu , C. C. Hsu , W. J. Lee , et al., “Muscle‐to‐Fat Ratio Identifies Functional Impairments and Cardiometabolic Risk and Predicts Outcomes: Biomarkers of Sarcopenic Obesity,” Journal of Cachexia, Sarcopenia and Muscle 13 (2022): 368–376. 34866342 10.1002/jcsm.12877 PMC8818605

[6] W. Wang , Y. Luo , Z. Zhuang , et al., “Total and Regional Fat‐to‐Muscle Mass Ratio and Risks of Incident All‐Cause Dementia, Alzheimer's Disease, and Vascular Dementia,” Journal of Cachexia, Sarcopenia and Muscle 13 (2022): 2447–2455. 35856185 10.1002/jcsm.13054 PMC9530585

[7] N. Wang , Y. Sun , H. Zhang , et al., “Total and Regional fat‐to‐Muscle Mass Ratio Measured by Bioelectrical Impedance and Risk of Incident Type 2 Diabetes,” Journal of Cachexia, Sarcopenia and Muscle 12 (2021): 2154–2162. 34595832 10.1002/jcsm.12822 PMC8718017

[8] R. Zhou , H. W. Chen , Y. Lin , et al., “Total and Regional Fat/Muscle Mass Ratio and Risks of Incident Cardiovascular Disease and Mortality,” Journal of the American Heart Association 12 (2023): e030101. 37642038 10.1161/JAHA.123.030101 PMC10547339

[9] B. Yu , Y. Sun , X. Du , et al., “Age‐Specific and sex‐Specific Associations of Visceral Adipose Tissue Mass and Fat‐to‐Muscle Mass Ratio With Risk of Mortality,” Journal of Cachexia, Sarcopenia and Muscle 14 (2023): 406–417. 36447372 10.1002/jcsm.13142 PMC9891960

[10] M. Xu , Y. Gong , and X. Yin , “Total and Regional Fat‐To‐Muscle Mass Ratio in Relation to All‐Cause and Cause‐Specific Mortality in Men and Women,” Journal of Clinical Endocrinology and Metabolism 110 (2025): e2054–e2063. 39193721 10.1210/clinem/dgae595

[11] M. Y. Ou , H. Zhang , P. C. Tan , S. B. Zhou , and Q. F. Li , “Adipose Tissue Aging: Mechanisms and Therapeutic Implications,” Cell Death & Disease 13 (2022): 300. 35379822 10.1038/s41419-022-04752-6 PMC8980023

[12] G. H. Li , G. K. Lee , P. C. Au , et al., “The Effect of Different Measurement Modalities in the Association of Lean Mass With Mortality: A Systematic Review and Meta‐Analysis,” Osteoporosis and Sarcopenia 7 (2021): S13–s18. 33997304 10.1016/j.afos.2021.02.004 PMC8088995

[13] J. Kim , Z. Wang , S. B. Heymsfield , R. N. Baumgartner , and D. Gallagher , “Total‐Body Skeletal Muscle Mass: Estimation by a New Dual‐Energy X‐ray Absorptiometry Method,” American Journal of Clinical Nutrition 76 (2002): 378–383. 12145010 10.1093/ajcn/76.2.378

[14] Y. Wu , T. Zhang , S. Li , J. Leung , and T. Kwok , “Utility of biological aging markers for mortality risk stratification in community‐dwelling older adults: Insights from the Mr. OS & Ms. OS (Hong Kong) cohort,” Journals of Gerontology. Series A, Biological Sciences and Medical Sciences 81 (2026): glag073. 41830155 10.1093/gerona/glag073 PMC13070468

[15] R. A. Washburn , K. W. Smith , A. M. Jette , and C. A. Janney , “The Physical Activity Scale for the Elderly (PASE): development and evaluation,” Journal of Clinical Epidemiology 46 (1993): 153–162. 8437031 10.1016/0895-4356(93)90053-4

[16] M. L. Mongraw‐Chaffin , S. A. E. Peters , R. R. Huxley , and M. Woodward , “The Sex‐Specific Association Between BMI and Coronary Heart Disease: A Systematic Review and Meta‐Analysis of 95 Cohorts With 1·2 Million Participants,” Lancet Diabetes and Endocrinology 3 (2015): 437–449. 25960160 10.1016/S2213-8587(15)00086-8 PMC4470268

[17] C. Huang , W. Liu , X. Ren , et al., “Association Between Human Herpesvirus 6 (HHV‐6) and Cognitive Function in the Elderly Population in Shenzhen, China,” Aging Clinical and Experimental Research 34 (2022): 2407–2415. 35767152 10.1007/s40520-022-02170-4

[18] J. J. Baechle , N. Chen , P. Makhijani , S. Winer , D. Furman , and D. A. Winer , “Chronic Inflammation and the Hallmarks of Aging,” Molecular Metabolism 74 (2023): 101755. 37329949 10.1016/j.molmet.2023.101755 PMC10359950

[19] L. Wu , M. Wu , D. Zhao , et al., “Elevated High‐Sensitivity C‐Reactive Protein Levels Increase the Risk of New‐Onset Cardiac Conduction Disorders,” Cardiovascular Diabetology 22 (2023): 268. 37777746 10.1186/s12933-023-01987-1 PMC10543876

[20] T. A. Pearson , G. A. Mensah , R. W. Alexander , et al., “Markers of Inflammation and Cardiovascular Disease: Application to Clinical and Public Health Practice: A Statement for Healthcare Professionals From the Centers for Disease Control and Prevention and the American Heart Association,” Circulation 107 (2003): 499–511. 12551878 10.1161/01.cir.0000052939.59093.45

[21] S. Duan , Y. Wu , J. Zhu , et al., “Development of Interpretable Machine Learning Models Associated With Environmental Chemicals to Predict All‐Cause and Specific‐Cause Mortality: A Longitudinal Study Based on NHANES,” Ecotoxicology and Environmental Safety 270 (2024): 115864. 38142591 10.1016/j.ecoenv.2023.115864

[22] T. E. Dorner and A. Rieder , “Obesity Paradox in Elderly Patients With Cardiovascular Diseases,” International Journal of Cardiology 155 (2012): 56–65. 21345498 10.1016/j.ijcard.2011.01.076

[23] F. D. C. De Stefani , P. S. Pietraroia , M. M. Fernandes‐Silva , J. Faria‐Neto , and C. P. Baena , “Observational Evidence for Unintentional Weight Loss in All‐Cause Mortality and Major Cardiovascular Events: A Systematic Review and Meta‐Analysis,” Scientific Reports 8 (2018): 15447. 30337578 10.1038/s41598-018-33563-z PMC6194006

[24] E. Cava , N. C. Yeat , and B. Mittendorfer , “Preserving Healthy Muscle During Weight Loss,” Advances in Nutrition 8 (2017): 511–519. 28507015 10.3945/an.116.014506 PMC5421125

[25] A. Jaitovich and J. B. Hall , “The Flux of Energy in Critical Illness and the Obesity Paradox,” Physiological Reviews 105 (2025): 1487–1552. 39982115 10.1152/physrev.00029.2024 PMC12107714

[26] K. N. Manolopoulos , F. Karpe , and K. N. Frayn , “Gluteofemoral Body Fat as a Determinant of Metabolic Health,” International Journal of Obesity 34 (2010): 949–959. 20065965 10.1038/ijo.2009.286

[27] N. Ouchi , J. L. Parker , J. J. Lugus , and K. Walsh , “Adipokines in inflammation and Metabolic Disease,” Nature Reviews. Immunology 11 (2011): 85–97. 10.1038/nri2921 PMC3518031 21252989

[28] C. K. Liang , L. N. Peng , M. H. Lin , et al., “Long‐Term Mortality Risk in Older Adults With Sarcopenia: An 11‐Year Prospective Cohort Study Comparing AWGS 2014 and AWGS 2019 Guidelines for Enhanced Clinical Utility and Accurate Risk Prediction,” Journal of Nutrition, Health & Aging 27 (2023): 507–513. 10.1007/s12603-023-1940-y PMC12929957 37498098

[29] H. Cooper , S. Wells , and S. Mehta , “Are Competing‐Risk Models Superior to Standard Cox Models for Predicting Cardiovascular Risk in Older Adults? Analysis of a Whole‐of‐Country Primary Prevention Cohort Aged ≥ 65 years,” International Journal of Epidemiology 51 (2022): 604–614. 34109395 10.1093/ije/dyab116

[30] S. Haldar , S. C. Chia , and C. J. Henry , “Body Composition in Asians and Caucasians: Comparative Analyses and Influences on Cardiometabolic Outcomes,” Advances in Food and Nutrition Research 75 (2015): 97–154. 26319906 10.1016/bs.afnr.2015.07.001

[31] I. A. Lesser , D. Gasevic , and S. A. Lear , “The Effect of Body Fat Distribution on Ethnic Differences in Cardiometabolic Risk Factors of Chinese and Europeans,” Applied Physiology, Nutrition, and Metabolism 38 (2013): 701–706. 10.1139/apnm-2012-0125 23980727

[32] K. E. Merz and D. C. Thurmond , “Role of Skeletal Muscle in Insulin Resistance and Glucose Uptake,” Compr Physiol 10 (2020): 785–809. 32940941 10.1002/cphy.c190029 PMC8074531

[33] K. S. Ruth , F. R. Day , J. Tyrrell , et al., “Using Human Genetics to Understand the Disease Impacts of Testosterone in Men and Women,” Nature Medicine 26 (2020): 252–258. 10.1038/s41591-020-0751-5 PMC7025895 32042192

[34] E. L. Watts , A. Perez‐Cornago , A. Knuppel , K. K. Tsilidis , T. J. Key , and R. C. Travis , “Prospective Analyses of Testosterone and Sex Hormone‐Binding Globulin With the Risk of 19 Types of Cancer in Men and Postmenopausal Women in UK Biobank,” International Journal of Cancer 149 (2021): 573–584. 33720423 10.1002/ijc.33555

[35] M. A. Argentieri , N. Amin , A. J. Nevado‐Holgado , et al., “Integrating the Environmental and Genetic Architectures of Aging and Mortality,” Nature Medicine 31 (2025): 1016–1025. 10.1038/s41591-024-03483-9 PMC11922759 39972219

[36] A. Jayedi , S. Soltani , M. S. Zargar , T. A. Khan , and S. Shab‐Bidar , “Central Fatness and Risk of All Cause Mortality: Systematic Review and Dose‐Response Meta‐Analysis of 72 Prospective Cohort Studies,” BMJ 370 (2020): m3324. 32967840 10.1136/bmj.m3324 PMC7509947

[37] H. Ahn , D. W. Kim , Y. Ko , et al., “Updated Systematic Review and Meta‐Analysis on Diagnostic Issues and the Prognostic Impact of Myosteatosis: A New Paradigm Beyond Sarcopenia,” Ageing Research Reviews 70 (2021): 101398. 34214642 10.1016/j.arr.2021.101398

[38] Y. Luo , L. Shu , Y. Wang , et al., “Sarcopenic Obesity and Risk of Cardio‐Cerebrovascular Disease and Mortality: A Systematic Review and Meta‐Analysis,” International Journal of Obesity 49 (2025): 2406–2414. 40947451 10.1038/s41366-025-01909-z

