# Supplementary material for: DXA‐Measured Total and Regional Fat‐to‐Lean Mass Ratio and Mortality Risk in Chinese Older Adults: A 20‐Year Prospective Study
Source: J Cachexia Sarcopenia Muscle. 2026 Jul 13;17(4):e70351. doi: 10.1002/jcsm.70351 (PMC13365363; doi:10.1002/jcsm.70351)
Supplement: Supplementary file 1 — Table S1: The survival rate of study sample in the Mr. OS & Ms. OS (Hong Kong) cohort. Table S2: Multivariable‐adjusted HRs (95% CIs) for all‐cause mortality by total and regional FLR with excluding those who died within 2 years after baseline survey using Cox regression model. Table S3: Multivariable‐adjusted HRs (95% CIs) for cause‐specific mortality by total and regional FLR with excluding those who died within 2 years after baseline survey using Fine–Gray competing risk model. Table S4: Multivariable‐adjusted HRs (95% CIs) for all‐cause mortality by total and regional fat‐to‐lean ratio using Cox regression model (adjusting hsCRP). Table S5: Multivariable‐adjusted HRs (95% CIs) for cause‐specific mortality by total and regional fat‐to‐lean ratio using Fine–Gray competing risk model (adjusting hsCRPP). Table S6: Multivariable‐adjusted HRs (95% CIs) for cancer‐specific mortality by total and regional fat‐to‐lean ratio using Fine–Gray competing risk model (excluding those who had incident cancer within the first 4‐year follow up). Figure S1: The Kaplan–Meier curves of all‐cause mortality stratified by total and regional fat‐to‐lean mass ratio levels in men. Figure S2: The Kaplan–Meier curves of all‐cause mortality stratified by total and regional fat‐to‐lean mass ratio levels in women. [file JCSM-17-e70351-s001.docx]

**DXA-measured total and regional fat-to-lean mass ratio and mortality risk in Chinese older adults: A 20-year prospective study**

Yafei Wu, Ting Zhang, Shuyi Li, Jason Leung, Timothy Kwok

**Table S1.** The survival rate of study sample in the Mr. OS & Ms. OS (Hong Kong) cohort

| Time | Number at risk | Number of events | Survival rate | Lower 95%CI | Upper 95%CI | Standard error |
| --- | --- | --- | --- | --- | --- | --- |
| Men | | | | | | |
| 1-year | 1981 | 19 | 0.990 | 0.986 | 0.995 | 0.00217 |
| 5-year | 1793 | 188 | 0.897 | 0.883 | 0.910 | 0.00681 |
| 10-year | 1506 | 287 | 0.753 | 0.734 | 0.772 | 0.00964 |
| 15-year | 1113 | 393 | 0.557 | 0.535 | 0.579 | 0.01111 |
| 20-year | 707 | 406 | 0.354 | 0.333 | 0.375 | 0.01069 |
| Women | | | | | | |
| 1-year | 1993 | 7 | 0.997 | 0.994 | 0.999 | 0.00132 |
| 5-year | 1920 | 73 | 0.960 | 0.951 | 0.969 | 0.00438 |
| 10-year | 1715 | 205 | 0.858 | 0.842 | 0.873 | 0.00782 |
| 15-year | 1417 | 298 | 0.708 | 0.689 | 0.729 | 0.01016 |
| 20-year | 881 | 416 | 0.500 | 0.479 | 0.523 | 0.01119 |

**Table S2.** Multivariable-adjusted HRs (95%CIs) for all-cause mortality by total and regional FLR with excluding those who died within two years after baseline survey using Cox regression model.

|  | Men | | Women | |
| --- | --- | --- | --- | --- |
|  | HR (95%CI) | *P*-value | HR (95%CI) | *P*-value |
| Whole body | | | | |
| Continuous | 0.95 (0.90-1.00) | 0.071 | 0.94 (0.88-1.01) | 0.077 |
| Tertile 1 | 1.17 (1.02-1.33) | 0.025 | 1.15 (0.99-1.34) | 0.076 |
| Tertile 2 | 1 (reference) | - | 1 (reference) | - |
| Tertile 3 | 1.10 (0.96-1.26) | 0.167 | 1.10 (0.95-1.29) | 0.209 |
| Trunk | | | | |
| Continuous | 0.93 (0.87-0.98) | 0.009 | 0.94 (0.88-1.00) | 0.065 |
| Tertile 1 | 1.27 (1.11-1.45) | 0.001 | 1.13 (0.97-1.32) | 0.107 |
| Tertile 2 | 1 (reference) | - | 1 (reference) | - |
| Tertile 3 | 1.11 (0.97-1.27) | 0.142 | 1.03 (0.89-1.20) | 0.701 |
| Abdominal |  |  |  |  |
| Continuous | 0.92 (0.87-0.98) | 0.010 | 0.92 (0.86-0.98) | 0.011 |
| Tertile 1 | 1.18 (1.02-1.35) | 0.021 | 1.17 (1.01-1.37) | 0.043 |
| Tertile 2 | 1 (reference) | - | 1 (reference) | - |
| Tertile 3 | 1.04 (0.91-1.19) | 0.566 | 1.00 (0.85-1.16) | 0.949 |
| Arm | | | | |
| Continuous | 0.95 (0.90-1.00) | 0.065 | 0.96 (0.91-1.03) | 0.261 |
| Tertile 1 | 1.23 (1.07-1.40) | 0.003 | 1.03 (0.88-1.20) | 0.720 |
| Tertile 2 | 1 (reference) | - | 1 (reference) | - |
| Tertile 3 | 1.12 (0.98-1.28) | 0.095 | 1.02 (0.88-1.19) | 0.789 |
| Leg | | | | |
| Continuous | 0.99 (0.94-1.05) | 0.739 | 0.94 (0.89-1.01) | 0.069 |
| Tertile 1 | 1.10 (0.96-1.26) | 0.156 | 1.15 (0.99-1.34) | 0.071 |
| Tertile 2 | 1 (reference) | - | 1 (reference) | - |
| Tertile 3 | 1.13 (0.99-1.29) | 0.073 | 1.02 (0.88-1.20) | 0.766 |

*Note*: All models were adjusted for age, educational level, smoking status, drinking, physical activity, diabetes, hypertension, cardiovascular disease, cancer, antihypertensive medication, cholesterol lowering medication, and total or regional absolute lean mass. FLR, fat-to-lean mass ratio. Results were reported by HR and 95%CI. For continuous FLR, HR per one standard deviation increase for each indicator was used. For male, 47 cases who died within 2 years after baseline survey were excluded. For female, 24 cases who died within the first 2 years after baseline survey were excluded.

**Table S3.** Multivariable-adjusted HRs (95% CIs) for cause-specific mortality by total and regional FLR with excluding those who died within two years after baseline survey using Fine-Gray competing risk model

|  | CVD-cause mortality | | | | Cancer-cause mortality | | | |
| --- | --- | --- | --- | --- | --- | --- | --- | --- |
|  | Men | | Women | | Men | | Women | |
|  | HR (95%CI) | *P*-value | HR (95%CI) | *P*-value | HR (95%CI) | *P*-value | HR (95%CI) | *P*-value |
| Whole body | | | | | | | | |
| Continuous | 1.06 (0.91-1.23) | 0.466 | 0.92 (0.77-1.09) | 0.323 | 0.93 (0.83-1.05) | 0.251 | 1.14 (0.98-1.33) | 0.080 |
| Tertile 1 | 0.89 (0.62-1.27) | 0.517 | 1.37 (0.94-2.01) | 0.105 | 1.01 (0.77-1.30) | 0.973 | 1.00 (0.71-1.42) | 0.981 |
| Tertile 2 | 1 (reference) | - | 1 (reference) | - | 1 (reference) | - | 1 (reference) | - |
| Tertile 3 | 1.13 (0.80-1.58) | 0.494 | 1.29 (0.87-1.91) | 0.200 | 0.95 (0.72-1.23) | 0.679 | 1.14 (0.82-1.58) | 0.438 |
| Trunk | | | | | | | | |
| Continuous | 1.04 (0.89-1.21) | 0.656 | 0.90 (0.76-1.07) | 0.246 | 0.96 (0.85-1.08) | 0.464 | 1.16 (0.99-1.35) | 0.067 |
| Tertile 1 | 1.04 (0.72-1.49) | 0.836 | 1.30 (0.90-1.88) | 0.168 | 1.17 (0.90-1.52) | 0.248 | 0.93 (0.66-1.31) | 0.689 |
| Tertile 2 | 1 (reference) | - | 1 (reference) | - | 1 (reference) | - | 1 (reference) | - |
| Tertile 3 | 1.17 (0.83-1.65) | 0.375 | 1.03 (0.70-1.52) | 0.889 | 1.14 (0.87-1.49) | 0.348 | 1.25 (0.90-1.72) | 0.179 |
| Abdominal |  |  |  |  |  |  |  |  |
| Continuous | 1.02 (0.87-1.20) | 0.811 | 0.90 (0.75-1.07) | 0.231 | 0.98 (0.87-1.11) | 0.780 | 1.09 (0.93-1.28) | 0.309 |
| Tertile 1 | 0.82 (0.57-1.18) | 0.287 | 1.31 (0.90-1.91) | 0.154 | 1.14 (0.87-1.49) | 0.338 | 0.91 (0.65-1.28) | 0.586 |
| Tertile 2 | 1 (reference) | - | 1 (reference) | - | 1 (reference) | - | 1 (reference) | - |
| Tertile 3 | 0.90 (0.64-1.26) | 0.544 | 1.07 (0.72-1.58) | 0.740 | 1.15 (0.88-1.50) | 0.316 | 1.00 (0.72-1.38) | 0.984 |
| Arm | | | | | | | | |
| Continuous | 1.07 (0.93-1.24) | 0.349 | 0.94 (0.79-1.11) | 0.440 | 0.93 (0.83-1.05) | 0.249 | 1.12 (0.97-1.29) | 0.133 |
| Tertile 1 | 0.96 (0.67-1.38) | 0.838 | 1.14 (0.79-1.64) | 0.483 | 1.15 (0.88-1.50) | 0.299 | 0.90 (0.64-1.26) | 0.521 |
| Tertile 2 | 1 (reference) | - | 1 (reference) | - | 1 (reference) | - | 1 (reference) | - |
| Tertile 3 | 1.17 (0.83-1.67) | 0.373 | 1.03 (0.70-1.53) | 0.866 | 1.12 (0.85-1.47) | 0.424 | 1.11 (0.81-1.54) | 0.519 |
| Leg | | | | | | | | |
| Continuous | 1.10 (0.96-1.25) | 0.172 | 0.97 (0.82-1.14) | 0.695 | 0.90 (0.80-1.00) | 0.058 | 1.07 (0.93-1.22) | 0.370 |
| Tertile 1 | 0.85 (0.60-1.21) | 0.360 | 1.24 (0.84-1.81) | 0.280 | 1.25 (0.97-1.62) | 0.089 | 1.13 (0.80-1.58) | 0.497 |
| Tertile 2 | 1 (reference) | - | 1 (reference) | - | 1 (reference) | - | 1 (reference) | - |
| Tertile 3 | 1.09 (0.77-1.53) | 0.625 | 1.23 (0.84-1.81) | 0.288 | 1.10 (0.84-1.44) | 0.507 | 1.16 (0.83-1.63) | 0.382 |

*Note*: All models were adjusted for age, educational level, smoking status, drinking, physical activity, diabetes, hypertension, cardiovascular disease (for cancer-cause), cancer (for CVD cause), antihypertensive medication, cholesterol lowering medication, and total or regional absolute lean mass. FLR, fat-to-lean mass ratio. Results were reported by HR and 95%CI. For continuous FLR, HR per one standard deviation increase for each FLR indicator was used. For CVD-cause mortality, the analytical sample sizes were 1513 for men and 1598 for women, after excluding those who had baseline CVD, had no death causes, and died within first two years. For cancer-cause mortality, the analytical sample sizes were 1865 for men and 1887 for women, after excluding those who had baseline cancer, had no death causes, and died within first two years.

**Table S4.** Multivariable-adjusted HRs (95% CIs) for all-cause mortality by total and regional fat-to-lean ratio using Cox regression model (adjusting hsCRP)

|  | Men | | Women | |
| --- | --- | --- | --- | --- |
|  | HR (95%CI) | *P*-value | HR (95%CI) | *P*-value |
| Whole body | | | | |
| Continuous | 0.93 (0.88-0.98) | 0.009 | 0.93 (0.87-0.99) | 0.026 |
| Tertile 1 | 1.21 (1.06-1.38) | 0.006 | 1.16 (1.00-1.36) | 0.051 |
| Tertile 2 | 1 (reference) | - | 1 (reference) | - |
| Tertile 3 | 1.08 (0.94-1.23) | 0.282 | 1.10 (0.94-1.28) | 0.222 |
| Trunk | | | | |
| Continuous | 0.90 (0.85-0.96) | 0.001 | 0.93 (0.87-0.99) | 0.022 |
| Tertile 1 | 1.27 (1.11-1.45) | <0.001 | 1.13 (0.98-1.32) | 0.104 |
| Tertile 2 | 1 (reference) | - | 1 (reference) | - |
| Tertile 3 | 1.06 (0.93-1.21) | 0.419 | 1.01 (0.87-1.17) | 0.922 |
| Abdominal |  |  |  |  |
| Continuous | 0.91 (0.85-0.96) | 0.001 | 0.90 (0.85-0.97) | 0.003 |
| Tertile 1 | 1.19 (1.04-1.37) | 0.010 | 1.18 (1.01-1.37) | 0.034 |
| Tertile 2 | 1 (reference) | - | 1 (reference) | - |
| Tertile 3 | 1.02 (0.89-1.16) | 0.806 | 0.97 (0.83-1.13) | 0.717 |
| Arm | | | | |
| Continuous | 0.93 (0.88-0.98) | 0.011 | 0.96 (0.90-1.02) | 0.161 |
| Tertile 1 | 1.26 (1.10-1.44) | 0.001 | 1.04 (0.89-1.21) | 0.641 |
| Tertile 2 | 1 (reference) | - | 1 (reference) | - |
| Tertile 3 | 1.10 (0.96-1.26) | 0.156 | 1.01 (0.87-1.17) | 0.934 |
| Leg | | | | |
| Continuous | 0.97 (0.92-1.03) | 0.341 | 0.94 (0.88-1.00) | 0.033 |
| Tertile 1 | 1.14 (1.00-1.30) | 0.053 | 1.16 (0.99-1.35) | 0.060 |
| Tertile 2 | 1 (reference) | - | 1 (reference) | - |
| Tertile 3 | 1.12 (0.98-1.27) | 0.098 | 1.01 (0.87-1.18) | 0.861 |

*Note*: All models were adjusted for age, educational level, smoking status, drinking, physical activity, diabetes, hypertension, cardiovascular disease, cancer, antihypertensive medication, cholesterol lowering medication, the total or regional absolute lean mass, and hsCRP (hsCRP data were available for 1411 of the 2000 men and 1407 of the 2000 women. To retain the full sample size and avoid excluding participants with missing information, we categorized CRP status using the clinical cut-off point of 3 mg/L. Participants were classified into three groups: CRP ≤ 3 mg/L, CRP > 3 mg/L, and missing hsCRP data). Results were reported by HR and 95%CI. For continuous FLR, HR per one standard deviation increase for each FLR indicator was used. FLR, fat-to-lean mass ratio; HR, hazard ratio; CI, confidence interval.

**Table S5.** Multivariable-adjusted HRs (95% CIs) for cause-specific mortality by total and regional fat-to-lean ratio using Fine-Gray competing risk model (adjusting hsCRPP)

|  | CVD-cause mortality | | | |  | Cancer-cause mortality | | | |
| --- | --- | --- | --- | --- | --- | --- | --- | --- | --- |
|  | Men | | Women | |  | Men | | Women | |
|  | HR (95%CI) | *P*-value | HR (95%CI) | *P*-value |  | HR (95%CI) | *P*-value | HR (95%CI) | *P*-value |
| Whole body | | | | |  |  | | | |
| Continuous | 1.06 (0.91-1.22) | 0.468 | 0.90 (0.75-1.06) | 0.206 |  | 0.90 (0.80-1.01) | 0.076 | 1.13 (0.97-1.31) | 0.108 |
| Tertile 1 | 0.91 (0.64-1.30) | 0.610 | 1.40 (0.96-2.05) | 0.082 |  | 1.05 (0.81-1.34) | 0.728 | 1.03 (0.74-1.44) | 0.866 |
| Tertile 2 | 1 (reference) | - | 1 (reference) | - |  | 1 (reference) | - | 1 (reference) | - |
| Tertile 3 | 1.15 (0.82-1.62) | 0.413 | 1.26 (0.85-1.86) | 0.246 |  | 0.92 (0.71-1.19) | 0.509 | 1.17 (0.85-1.61) | 0.340 |
| Trunk | | | | |  |  | | | |
| Continuous | 1.04 (0.89-1.21) | 0.637 | 0.88 (0.74-1.04) | 0.142 |  | 0.92 (0.82-1.03) | 0.162 | 1.15 (0.99-1.33) | 0.076 |
| Tertile 1 | 1.01 (0.71-1.44) | 0.953 | 1.33 (0.92-1.92) | 0.125 |  | 1.20 (0.93-1.55) | 0.155 | 0.91 (0.66-1.27) | 0.587 |
| Tertile 2 | 1 (reference) | - | 1 (reference) | - |  | 1 (reference) | - | 1 (reference) | - |
| Tertile 3 | 1.12 (0.79-1.59) | 0.514 | 1.00 (0.67-1.50) | 0.990 |  | 1.09 (0.83-1.42) | 0.547 | 1.22 (0.89-1.67) | 0.221 |
| Abdominal |  |  |  |  |  |  |  |  |  |
| Continuous | 1.02 (0.88-1.20) | 0.772 | 0.87 (0.73-1.05) | 0.139 |  | 0.95 (0.85-1.07) | 0.382 | 1.07 (0.91-1.25) | 0.409 |
| Tertile 1 | 0.82 (0.57-1.18) | 0.286 | 1.35 (0.93-1.95) | 0.117 |  | 1.14 (0.88-1.48) | 0.308 | 0.93 (0.67-1.30) | 0.675 |
| Tertile 2 | 1 (reference) | - | 1 (reference) | - |  | 1 (reference) | - | 1 (reference) | - |
| Tertile 3 | 0.91 (0.65-1.28) | 0.596 | 1.05 (0.71-1.56) | 0.816 |  | 1.07 (0.82-1.39) | 0.629 | 0.99 (0.72-1.36) | 0.961 |
| Arm | | | | |  |  |  |  |  |
| Continuous | 1.07 (0.92-1.23) | 0.378 | 0.91 (0.77-1.09) | 0.304 |  | 0.90 (0.81-1.01) | 0.086 | 1.11 (0.97-1.28) | 0.145 |
| Tertile 1 | 1.01 (0.71-1.44) | 0.949 | 1.17 (0.81-1.68) | 0.399 |  | 1.24 (0.96-1.60) | 0.106 | 0.88 (0.63-1.23) | 0.457 |
| Tertile 2 | 1 (reference) | - | 1 (reference) | - |  | 1 (reference) | - | 1 (reference) | - |
| Tertile 3 | 1.18 (0.83-1.68) | 0.347 | 1.02 (0.68-1.52) | 0.933 |  | 1.13 (0.86-1.47) | 0.378 | 1.08 (0.79-1.48) | 0.638 |
| Leg | | | | |  |  |  |  |  |
| Continuous | 1.10 (0.96-1.25) | 0.176 | 0.96 (0.82-1.12) | 0.594 |  | 0.88 (0.78-0.98) | 0.020 | 1.05 (0.92-1.20) | 0.494 |
| Tertile 1 | 0.84 (0.59-1.19) | 0.323 | 1.24 (0.83-1.79) | 0.261 |  | 1.32 (1.02-1.69) | 0.033 | 1.10 (0.79-1.53) | 0.579 |
| Tertile 2 | 1 (reference) | - | 1 (reference) | - |  | 1 (reference) | - | 1 (reference) | - |
| Tertile 3 | 1.09 (0.77-1.52) | 0.635 | 1.22 (0.83-1.79) | 0.305 |  | 1.08 (0.83-1.41) | 0.560 | 1.10 (0.79-1.52) | 0.581 |

*Note*: All models were adjusted for age, educational level, smoking status, drinking, physical activity, diabetes, hypertension, cardiovascular disease (for cancer-cause), cancer (for CVD-cause), antihypertensive medication, cholesterol lowering medications, total or regional fat-to-lean mass ratio, and hsCRP (hsCRP data were available for 1411 of the 2000 men and 1407 of the 2000 women. To retain the full sample size and avoid excluding participants with missing information, we categorized CRP status using the clinical cut-off point of 3 mg/L. Participants were classified into three groups: CRP ≤ 3 mg/L, CRP > 3 mg/L, and missing hsCRP data). Results were reported by HR and 95%CI. For continuous FLR (fat-to-lean ratio), HR per one standard deviation increase was used. HR, hazard ratio; CI, confidence interval. For CVD-cause analysis: in men, the analytical sample was 1541 (7 cases without death causes and 452 participants with baseline CVD were excluded); in women, the analytical sample size was 1618 (2 cases without death causes and 382 participants with baseline CVD were excluded). For Cancer-cause analysis: in men, the analytical sample was 1907 (7 cases without death causes and 86 participants with baseline cancer were excluded); in women, the analytical sample size was 1909 (2 cases without death causes and 89 participants with baseline cancer were excluded).

**Table S6.** Multivariable-adjusted HRs (95% CIs) for cancer-specific mortality by total and regional fat-to-lean ratio using Fine-Gray competing risk model (excluding those who had incident cancer within the first 4-year follow up)

|  | Men | | Women | |
| --- | --- | --- | --- | --- |
|  | HR (95%CI) | *P*-value | HR (95%CI) | *P*-value |
| Whole body | | | | |
| Continuous | 0.86 (0.74-1.00) | 0.043 | 1.14 (0.94-1.37) | 0.181 |
| Tertile 1 | 1.16 (0.85-1.59) | 0.357 | 1.08 (0.71-1.62) | 0.724 |
| Tertile 2 | 1 (reference) | - | 1 (reference) | - |
| Tertile 3 | 0.97 (0.70-1.35) | 0.860 | 1.20 (0.82-1.77) | 0.347 |
| Trunk | | | | |
| Continuous | 0.89 (0.77-1.03) | 0.133 | 1.15 (0.95-1.39) | 0.162 |
| Tertile 1 | 1.37 (0.99-1.89) | 0.060 | 0.95 (0.64-1.42) | 0.808 |
| Tertile 2 | 1 (reference) | - | 1 (reference) | - |
| Tertile 3 | 1.19 (0.85-1.67) | 0.306 | 1.19 (0.82-1.74) | 0.368 |
| Abdominal |  |  |  |  |
| Continuous | 0.91 (0.79-1.06) | 0.216 | 1.10 (0.90-1.34) | 0.354 |
| Tertile 1 | 1.32 (0.95-1.84) | 0.097 | 0.94 (0.63-1.41) | 0.767 |
| Tertile 2 | 1 (reference) | - | 1 (reference) | - |
| Tertile 3 | 1.25 (0.90-1.74) | 0.184 | 1.06 (0.72-1.55) | 0.776 |
| Arm | | | | |
| Continuous | 0.88 (0.75-1.02) | 0.087 | 1.14 (0.96-1.36) | 0.136 |
| Tertile 1 | 1.15 (0.84-1.58) | 0.377 | 0.86 (0.57-1.28) | 0.446 |
| Tertile 2 | 1 (reference) | - | 1 (reference) | - |
| Tertile 3 | 1.02 (0.72-1.43) | 0.928 | 1.14 (0.78-1.66) | 0.506 |
| Leg | | | | |
| Continuous | 0.82 (0.71-0.95) | 0.008 | 1.03 (0.88-1.22) | 0.690 |
| Tertile 1 | 1.32 (0.97-1.79) | 0.081 | 1.25 (0.85-1.85) | 0.259 |
| Tertile 2 | 1 (reference) | - | 1 (reference) | - |
| Tertile 3 | 1.03 (0.74-1.45) | 0.854 | 1.20 (0.80-1.78) | 0.381 |

*Note*: All models were adjusted for age, educational level, smoking status, drinking, physical activity, diabetes, hypertension, cardiovascular disease, antihypertensive medication, cholesterol lowering medications, and total or regional absolute lean mass. Results were reported by HR and 95%CI. For continuous FLR (fat-to-lean ratio), HR per one standard deviation increase was used. HR, hazard ratio; CI, confidence interval. For men, the analytical sample was 1445 (7 cases without death causes, 86 participants with baseline cancer, 56 with incident cancer, and 406 lost to follow-up were excluded). For women, the analytical sample size was 1493 (2 cases without death causes, 89 participants with baseline cancer, 26 with incident cancer, and 390 lost to follow-up were excluded).

**
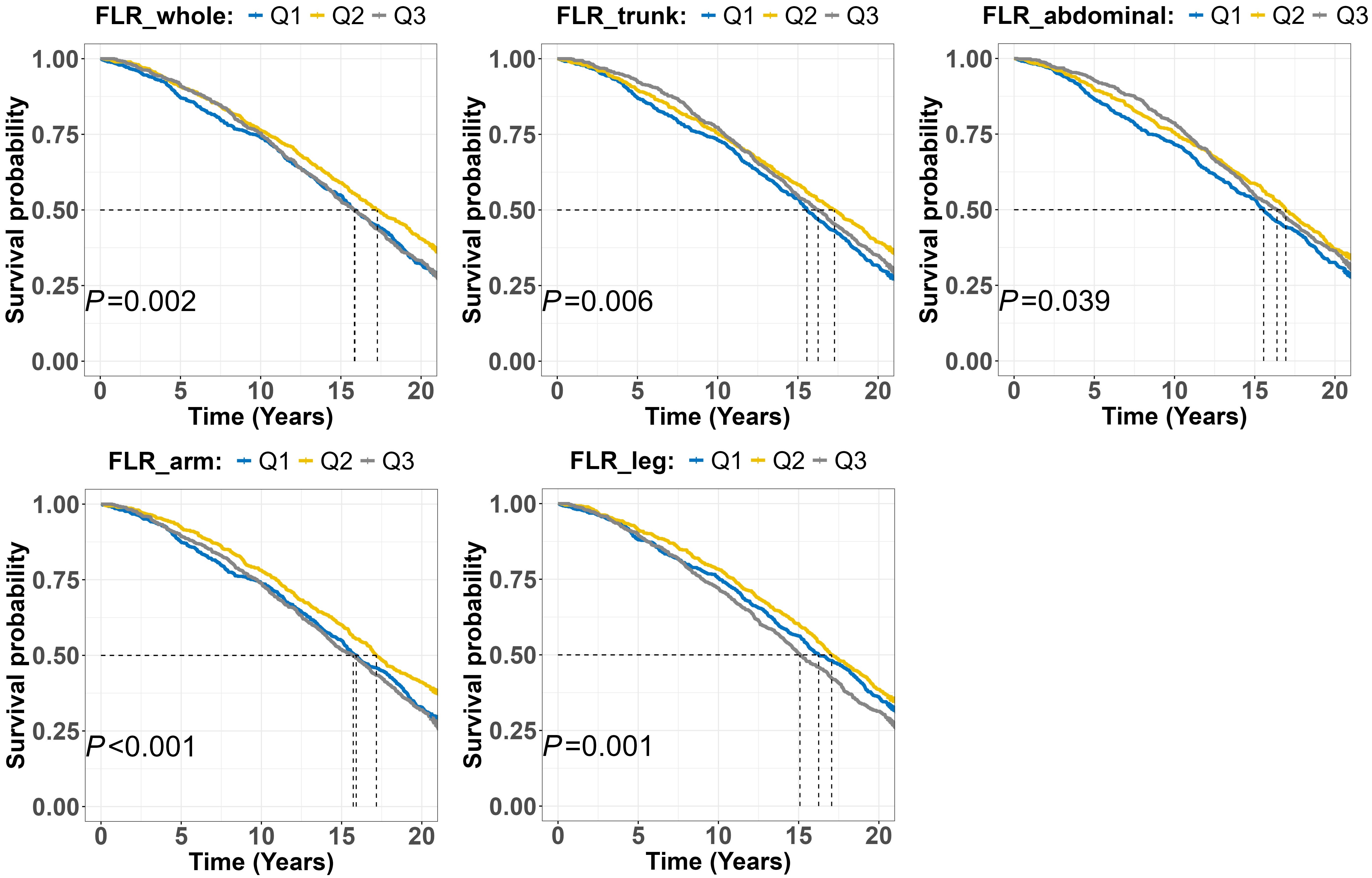
**

**Figure S1.** The Kaplan-Meier curves of all-cause mortality stratified by total and regional fat-to-lean mass ratio levels in men. Q1-Q3 in the figure denote tertile 1-tertile 3.


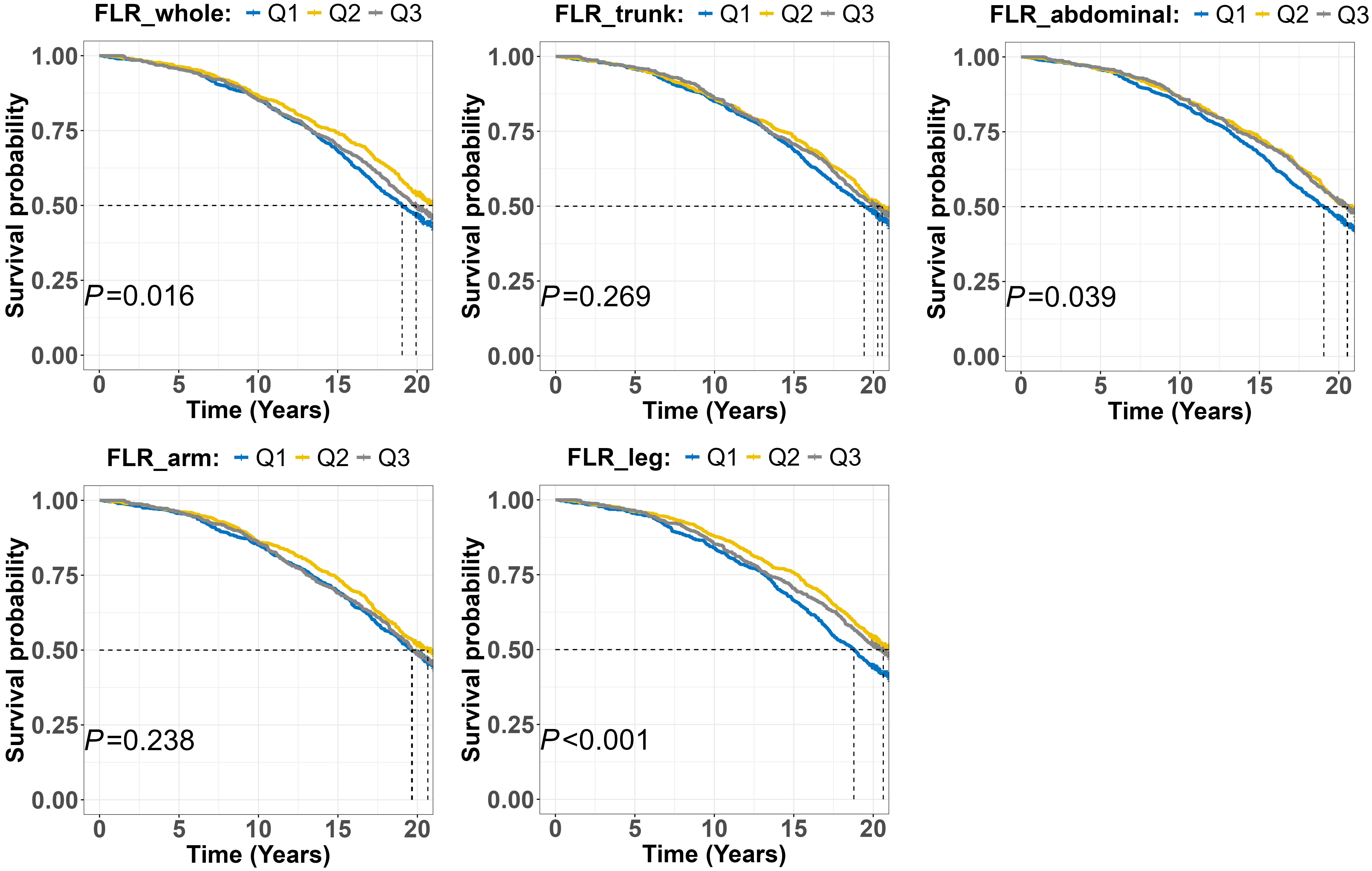


**Figure S2.** The Kaplan-Meier curves of all-cause mortality stratified by total and regional fat-to-lean mass ratio levels in women. Q1-Q3 in the figure denote tertile 1-tertile 3.
